# Pupillary light reflex circuits in the macaque monkey: the preganglionic Edinger–Westphal nucleus

**DOI:** 10.1007/s00429-019-02000-w

**Published:** 2019-12-24

**Authors:** Paul J. May, Wensi Sun, Nicholas F. Wright, Jonathan T. Erichsen

**Affiliations:** 1 Department of Neurobiology and Anatomical Sciences, University of Mississippi Medical Center, Jackson, 39216 MS USA; 2 Department of Ophthalmology, University of Mississippi Medical Center, Jackson, 39216 MS USA; 3 Department of Neurology, University of Mississippi Medical Center, Jackson, 39216 MS USA; 4 School of Optometry and Vision Sciences, Cardiff University, Maindy Road, Cardiff, Wales CF24 4HQ UK; 5 Present Address: North Gate Eye Center, 10564 5th Avenue Northeast, Suite 102, Seattle, WA 98125 USA; 6 Present Address: Nanopharm Ltd, Cavendish House, Newport, NP10 8FY UK

**Keywords:** Pupil, Autonomic, Luminance, Midbrain, Near response

## Abstract

The motor outflow for the pupillary light reflex originates in the preganglionic motoneuron subdivision of the Edinger–Westphal nucleus (EWpg), which also mediates lens accommodation. Despite their importance for vision, the morphology, ultrastructure and luminance-related inputs of these motoneurons have not been fully described in primates. In macaque monkeys, we labeled EWpg motoneurons from ciliary ganglion and orbital injections. Both approaches indicated preganglionic motoneurons occupy an EWpg organized as a unitary, ipsilateral cell column. When tracers were placed in the pretectal complex, labeled terminals targeted the ipsilateral EWpg and reached contralateral EWpg by crossing both above and below the cerebral aqueduct. They also terminated in the lateral visceral column, a ventrolateral periaqueductal gray region containing neurons projecting to the contralateral pretectum. Combining olivary pretectal and ciliary ganglion injections to determine whether a direct pupillary light reflex projection is present revealed a labeled motoneuron subpopulation that displayed close associations with labeled pretectal terminal boutons. Ultrastructurally, this subpopulation received synaptic contacts from labeled pretectal terminals that contained numerous clear spherical vesicles, suggesting excitation, and scattered dense-core vesicles, suggesting peptidergic co-transmitters. A variety of axon terminal classes, some of which may serve the near response, synapsed on preganglionic motoneurons. Quantitative analysis indicated that pupillary motoneurons receive more inhibitory inputs than lens motoneurons. To summarize, the pupillary light reflex circuit utilizes a monosynaptic, excitatory, bilateral pretectal projection to a distinct subpopulation of EWpg motoneurons. Furthermore, the interconnections between the lateral visceral column and olivary pretectal nucleus may provide pretectal cells with bilateral retinal fields.

## Introduction

The preganglionic motoneurons whose axons travel with the third cranial nerve are located in the preganglionic subdivision of the Edinger–Westphal nucleus (EWpg) (Kozicz et al. 2011; May et al. 2008a), a cell group associated with the oculomotor nucleus (III) that was first described by Edinger (1885) and Westphal (1887) over a century ago. These parasympathetic motoneurons synapse on postganglionic motoneurons in the ciliary ganglion that, in turn, supply the intraocular muscles of the eye. There are two populations within EWpg: one controls lens accommodation by activating the ciliary muscle and the other controls pupillary constriction by activating the pupillary sphincter muscle (Gamlin et al. 1984, 1994; Hultborn et al. 1973; McDougal and Gamlin 2015; May et al. 2019b).

Two behaviors use this parasympathetic outflow: the near response and the pupillary light reflex. The near response is initiated when animals direct their eyes to a nearby object. To do this, they execute three interrelated actions. (1) The lines of sight are rotated nasally (converged), pointing the foveae of both eyes toward the object. (2) The curvature of the lens is increased through the actions of the ciliary muscle, to focus the closer object. (3) The pupils constrict to produce greater depth of focus and less spherical aberration. The simultaneous occurrence of accommodation, convergence and pupillary constriction form the near triad. The pupillary light reflex acts to regulate the amount of light falling on the retina to optimize luminance levels for the photoreceptors. The short latency response of the pupil is primarily regulated by the parasympathetic input to the iris (Loewenfeld 1993). The pathway for the pupillary light reflex originates with retinal ganglion cells that can be characterized as broad-field luminance detectors. These cells contain melanopsin and have been characterized as intrinsically photoreceptive retinal ganglion cells (ipRGCs), although they also receive photoreceptor input (Güler et al. 2008; Hannibal et al. 2014). They send their axons to the olivary pretectal nucleus (OPt), with the temporal retina projecting to the ipsilateral OPt and the nasal retina providing input via the chiasm to the contralateral OPt. Like their retinal inputs, the cells in OPt are also classified as broad-field luminance detectors (Gamlin et al. 1995). This nucleus is believed to subsequently project to the EWpg. The action of this pathway is inhibited when there is increased activation of sympathetic pathways to the pupillary dilator muscle under low illumination conditions and due to changes in the state of the animal. In the latter, the pupils dilate in response to increased states of attention, arousal and/or cognitive load (Kahneman and Beatty 1966; Beatty 1982; Gabay et al. 2011; Szabadi 2013; Joshi et al. 2016), as well as with saccades (Wang and Munoz 2015).

Despite the critical role of EWpg motoneurons in these two important visual functions, there is relatively little information available about their inputs and ultrastructure in the primate, compared to other mammalian species (Ichinohe et al. 1996; Klooster et al. 1995; Sun and May 2014a, b). Even the organization of the primate EWpg is a matter of dispute. Some authorities describe this nucleus as a unitary column stretching rostrocaudally, dorsal to III (Akert et al. 1980; May et al. 2008a), whereas others have divided the nucleus into a number of subdivisions (Burde 1983; Burde and Williams 1989). In fact, it has been suggested that the pupillary preganglionic motoneurons inhabit one of these subdivisions, the lateral visceral column (Burde 1983; Büttner-Ennever et al. 1996; Kourouyan and Horton 1997). The precise pattern of connections between the pretectum and EWpg is also a matter of dispute. Some believe that OPt projects bilaterally, with decussating fibers traveling in the posterior commissure (Benevento et al. 1977; Klooster et al. 1995; Kourouyan and Horton 1997). However, other reports indicate a strictly contralateral projection (Steiger and Büttner-Ennever 1979; Clarke et al. 1985a). In fact, a monosynaptic projection of the pretectum onto preganglionic motoneurons has not been proven in primates.

To fill these gaps in our knowledge, we have undertaken a comprehensive characterization of these preganglionic parasympathetic motoneurons and their synaptic contacts in the macaque monkey. Furthermore, we have utilized neuronal tracers to label synaptic input from the pretectum to discriminate motoneurons involved in pupillary constriction. Brief reports of some of these data have appeared previously (Sun and May 1995; Erichsen et al. 1998; May et al. 2008b).

## Methods

The surgical procedures described below are in accordance with NIH guidelines and were approved by the Institutional Animal Care and Use Committee of the University of Mississippi Medical Center. Material from *Macaca fascicularis* (*n* = 13) and *Macaca mulatta* (*n* = 5) monkeys (> 3.0 kg) of both sexes was used in this study. Some of these cases were also used in other, non-conflicting studies. The animals were initially sedated with ketamine HCl (10 mg/kg, IM). For intraocular injections, animals were anesthetized with ketamine (22 mg/kg, IM) and xylazine (1 mg/kg, IM), and then proparacaine drops were applied to the cornea. For central injections and injections of the ganglia, an i.v. line was put in place to maintain hydration and a tracheal tube was introduced to allow induction of inhalation anesthesia with 3% isoflurane. Vital signs including temperature and heart rate were monitored and maintained within normal limits. The animals were given dexamethasone (1 mg/kg, IV) and atropine sulfate (0.05 mg/kg, IV) to control brain edema and respiratory secretions, respectively. All surgical procedures took place in a surgical suite and utilized sterile technique. The animals received Butorphanol (0.01 mg/kg, IM) or Buprenex (0.001 mg/kg, IM) as a postsurgical analgesic. Before cardiac perfusion, they were sedated with ketamine HCl (10 mg/kg, IM) and then deeply anesthetized with sodium pentobarbital (50 mg/kg, IP).

### Injections

To trans-synaptically label preganglionic motoneurons, injections of 2% wheat germ agglutinin (WGA) (Sigma) or 2% WGA conjugated to horseradish peroxidase (WGA–HRP) (Sigma) were placed either in the vitreous chamber or the aqueous of the anterior chamber of the eye (see Table 1 for details). The tracer was held in a 50-µl Hamilton syringe equipped with a 25G needle. The syringe needle was advanced through the cornea for anterior chamber injections and through the conjunctiva for vitreal injections. In some cases, the anterior chamber was injected on one side and the vitreous was injected on the other with the goal of differentially labeling pupillary circuits (Erichsen and May 2002). However, no indication of such specificity was observed in the ciliary ganglion and EWpg. A 4.0% paraformaldehyde fixative in 0.1 M, pH 7.2 phosphate buffer (PB) was used for animals with WGA injections, and a 1.0% paraformaldehyde, 1.25% glutaraldehyde fixative in 0.1 M, pH 7.2 PB was used for the WGA–HRP animals.

**Table 1:** Case injection details

Target	Tracer	Cases	Site #	Amount/site	Survival
Anterior	2% WGA	2	n.a.	25–50 µl	4 days
L. anterior R. vitreous	2% WGA–HRP	4	n.a.	25–50 µl	4 days
Ciliary ganglion	WGA–HRP/HRP	2^a^	n.a.	n.a.	1 day
Pretectum	1% WGA–HRP	1	3	0.03 µl	1 day
Pretectum	4% Biocytin	2^a^	2–3	0.2 µl	1 day
Pretectum	10% BDA	4	− 2	0.1–0.4 µl	21 days
Pretectum	2.5% PhaL	2	1	n.a.	14 days

*n.a.* not applicable

^a^One case had both a ciliary ganglion and pretectal injection

We used a lateral approach to inject the ciliary ganglion. An incision was made in the skin of the left temple. The anterior edge of the temporalis muscle was disinserted and retracted caudally. The skull over the lateral caudal aspect of the orbit was removed to allow visualization of the lateral rectus muscle, which was disinserted from the globe and retracted. A syringe with a 27 G needle was used to remove aqueous from the anterior chamber to partially deflate the globe. Blunt dissection revealed the optic nerve, and then the short ciliary nerves, which accompany it, were followed back to the ciliary ganglion. Connective tissue within this ganglion makes it difficult to inject using a needle. Therefore, we utilized small insect pins mounted on orange sticks to place the tracer in the ganglion. A paste of WGA–HRP and HRP was dried onto the pin tips. These were inserted into the ganglion and held until the tracer dissolved. The muscles were reattached and the overlying skin edges sutured together. One of these animals also received a pretectal biocytin injection, as described below.

A number of different tracers were used to label pretectal inputs to the EWpg: WGA–HRP, biocytin, biotinylated dextran amine (10,000 MW) (BDA) and *Phaseolus vulgaris* leukoagglutinin (PhaL) (see Table 1 for details). Due to the small size of the olivary pretectal nucleus (OPt), this nucleus was not injected in isolation. The dorsal surface of the midbrain was visualized by aspirating the overlying cortex. PhaL was injected iontophoretically using a glass micropipette with a 25-µm tip (7 µA, for 10 min, 50% duty cycle positive current). The other tracers were injected using a 1-µl Hamilton syringe. In each case, the needle or pipette was angled between 23^o^ and 30^o^ tip up from vertical in the parasagittal plane. The defect produced by the aspiration was filled with Gelfoam and the incision closed. After the appropriate survival times, the animals were deeply anesthetized and then perfused with buffered saline followed by 1.0% paraformaldehyde and 1.25% glutaraldehyde in 0.1 M, pH 7.2 PB (WGA–HRP and BDA) or 2.0% paraformaldehyde, 1.0% glutaraldehyde in 0.1 M, pH 7.2 PB (Biocytin and PhaL).

### Histological procedures

After perfusion, the brainstem was blocked in the stereotaxic frontal plane, removed and postfixed in the fixative solution for 2 h. It was then stored in phosphate buffer at 4 °C until it could be cut and processed. The brainstem was cut into 50- or 100-μm sections in the frontal plane by use of a vibratome (Leica). Alternatively, the samples were cryoprotected in 30% sucrose and frozen sectioned on a sliding microtome (AO) at 40 or 80 μm. Ordered 1 in 3 series were reacted to reveal the tracers. Those tagged with HRP were reacted using the tetramethylbenzidine (TMB) procedure of Olucha et al. (1985) (see Perkins et al. 2009 for details). In other cases, we used the nitroprusside TMB method (Mesulam 1978). To reveal tracers tagged with biotin (Biocytin and BDA), the tissue was reacted with avidin-conjugated horseradish peroxidase (avidin–HRP) (Vector Labs) and then the HRP was revealed with the chromogen diaminobenzidine (DAB) using the procedure of Adams (1977) (see Perkins et al. 2009 for details). To visualize the PhaL, we used the method of Gerfen and Sawchenko (1984) (see Wang et al. 2013 for details) that uses biotinylated goat anti-PhaL (Vector Labs) and a goat ABC kit (Vector Labs). The HRP was visualized by use of DAB, as described above. For the dual-tracer experiment, the TMB protocol of Olucha was followed, and the blue reaction product was protected by DAB to reveal retrogradely transported WGA–HRP, followed by the biocytin protocol that used DAB intensified with nickel and cobalt (see Perkins et al. 2009 for details).

For light microscopy, the sections were mounted, counterstained with cresyl violet or neutral red, dehydrated, cleared and coverslipped. For electron microscopy (EM), areas containing labeled preganglionic motoneurons were excised under visual control using a Wild M8 stereomicroscope and prepared for EM. Care was taken to exclude the adjacent oculomotor nucleus, which contained scattered labeled motoneurons due to the spread of tracer from the ganglion. The sections were then prepared for light microscopy to allow sample area verification. The EM samples were processed and cut for EM using standard procedures (Barnerssoi and May 2016). Semithin sections were taken from the block face, stained with Toluidine, and used to direct further trimming to the area of interest. Ultrathin sections were photographed with a Zeiss 10C or a Leo transmission electron microscope. Synaptic contacts were photographed at a standard magnification of 21,560X on the EM, but the magnification for somata and dendrites varied.

### Quantitative analysis of electron micrographs

The original electron micrographs were scanned to JPEG images and the terminals classified. Terminal classification, based on vesicle shape and synaptic density, and terminal measurements were made by separate individuals who were blinded to each other’s findings. The JPEG files were converted into tagged image format (TIF) to enable the use of NIH Image for Macintosh to process the images [https:/imagej.nih.gov/nih-image/download.html]. The images were first scaled to provide the correct number of pixels representing each micrometer. Then a ‘markup’ macro provided with NIH Image was loaded and a sufficient number of distinct colors were reserved in the look-up table (LUT) to allow the required number of image features to be traced with different colors. Boundaries of axon terminals, dendrites and cell bodies were marked onto the image along with lengths of membrane contacted by profiles and by synapses. Once colored graphics were extracted for measurement, NIH Image was used to ‘density slice’ through the LUT colors and separately measure the cross-sectional area, perimeter, major and minor axes, as well as the length of profile contact and synaptic contact. Where spines were visible, their values were not included in the ‘true’ profile and synaptic totals. The numerical data produced were saved to a spreadsheet and subsequently analyzed. Data were compared using a Student’s *T* test, and significance was set at *p* values less than 0.05.

## Results

### Preganglionic motoneurons

**Fig. 1:**
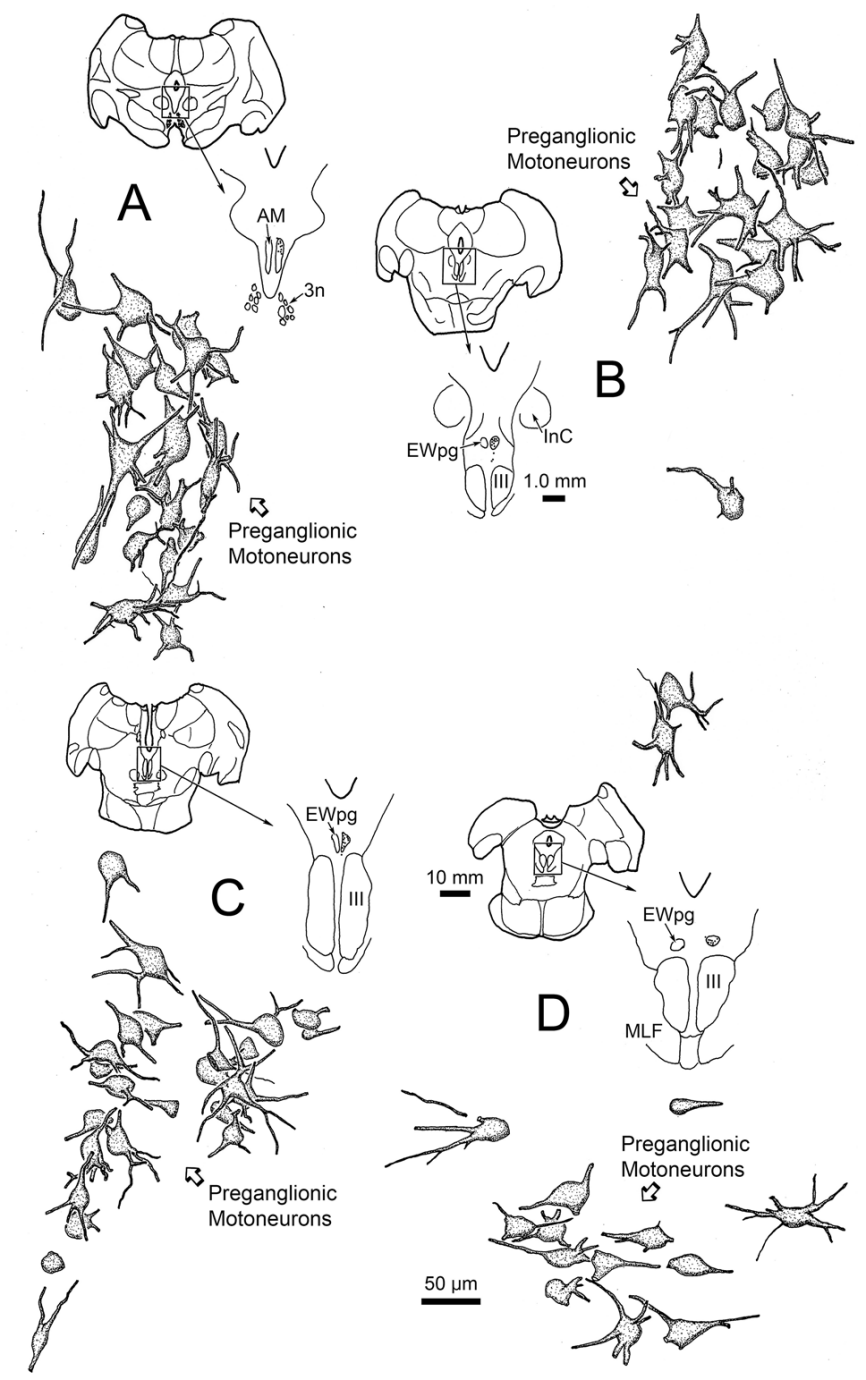
Retrogradely labeled preganglionic motoneurons in the Edinger–Westphal nucleus of the macaque monkey. Following a ciliary ganglion injection, labeled cells were observed throughout the ipsilateral EWpg (**b**–**d**) and extended rostrally into the AM (**a**). Most displayed multipolar morphology. Note that somatic motoneuron labeling was present, but is not illustrated here. Insets show section illustrated and region around III

**Fig. 2:**
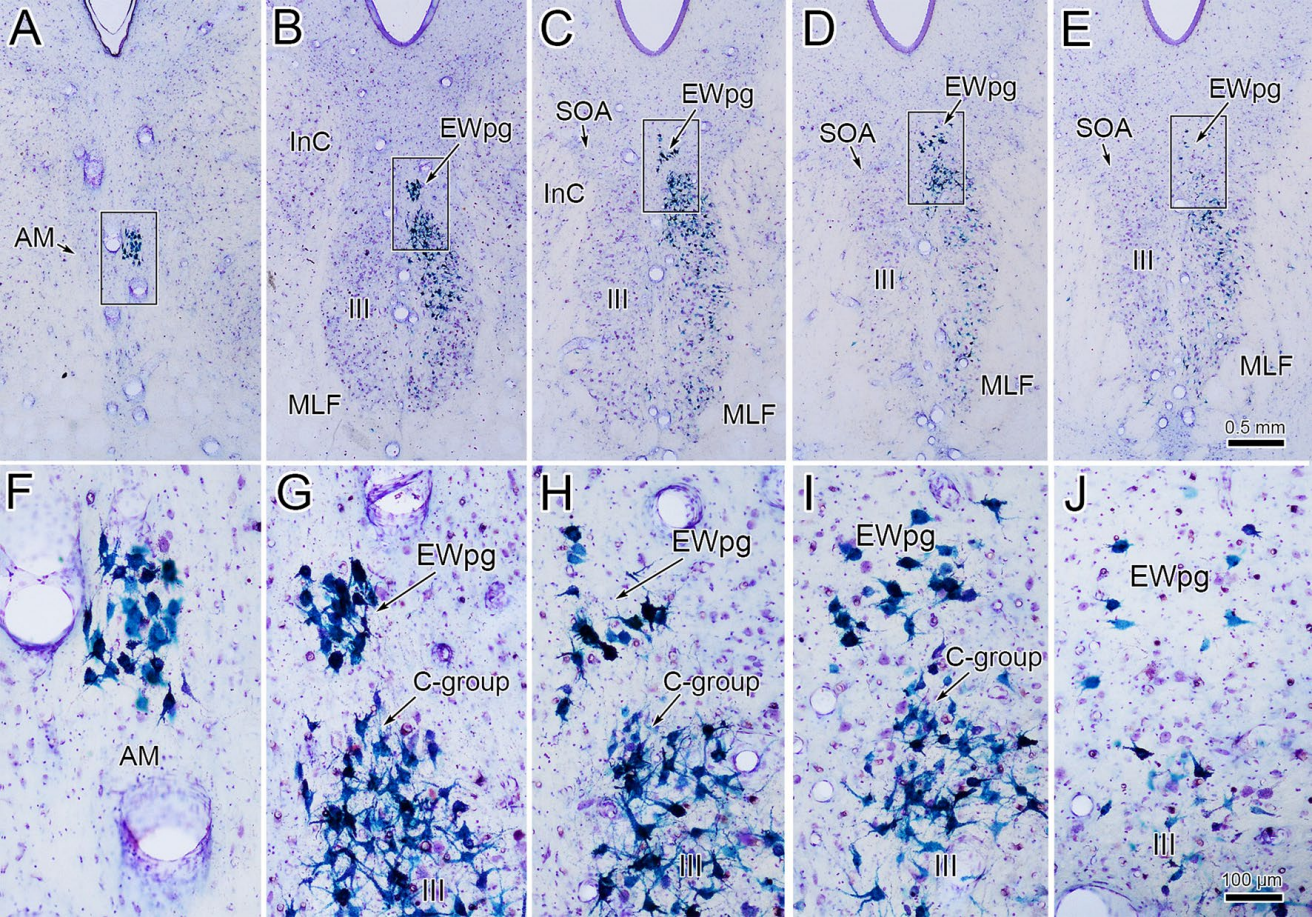
Appearance of retrograde labeling in preganglionic motoneurons following ciliary ganglion injection (same case as Fig. 1). **a–e** Rostral to caudal series of low-magnification views to provide context. The boxed areas are shown at higher magnification (**f–j**). The blue reaction product intensely labels the motoneurons in EWpg. Note that, as one proceeds caudally, the preganglionic motoneurons become more dispersed. Reaction product is also observed within extraocular motoneurons within III and within C-group. Scale in **ea**–**d**, **j** = **f**–**i**

WGA–HRP/HRP injections to the right ciliary ganglion produced labeling in the ipsilateral preganglionic motoneuron population. As shown in Fig. 1, the retrograde label filled the somata and proximal dendrites of the motoneurons located in a relatively tight cluster dorsal to the oculomotor nucleus (III) (Fig. 1b–d) and extending into the anteromedian nucleus (AM) rostral to III (Fig. 1a). The low- (a–e) and high-magnification (f–j) images of Fig. 2 provide a wider view of the labeling pattern. Retrogradely labeled, multipolar neurons formed an ipsilateral column that began in AM (Fig. 2a, f). They formed a tight cluster as the EWpg, which was located in the supraoculomotor area (SOA) dorsal to III at its rostral end (Fig. 2b, g). As one proceeds more caudally, the labeled cells became increasingly more dispersed (Fig. 2h–j) and moved slightly laterally at the caudal end (Fig. 1d). Spread of the tracer into the adjacent muscles produced somatic motoneuron labeling as well (Fig. 2b–d), including labeling of C-group motoneurons (Fig. 2g–h) that are known to supply input to multiply innervated muscle fibers (Büttner-Ennever and Akert 1981; Wasicky et al. 2004). At rostral levels, a few scattered labeled cells were observed ventral to EWpg (Fig. 1b). Since there was also labeling of extraocular motoneurons, these may represent C-group cells or more scattered preganglionic motoneurons.

**Fig. 3:**
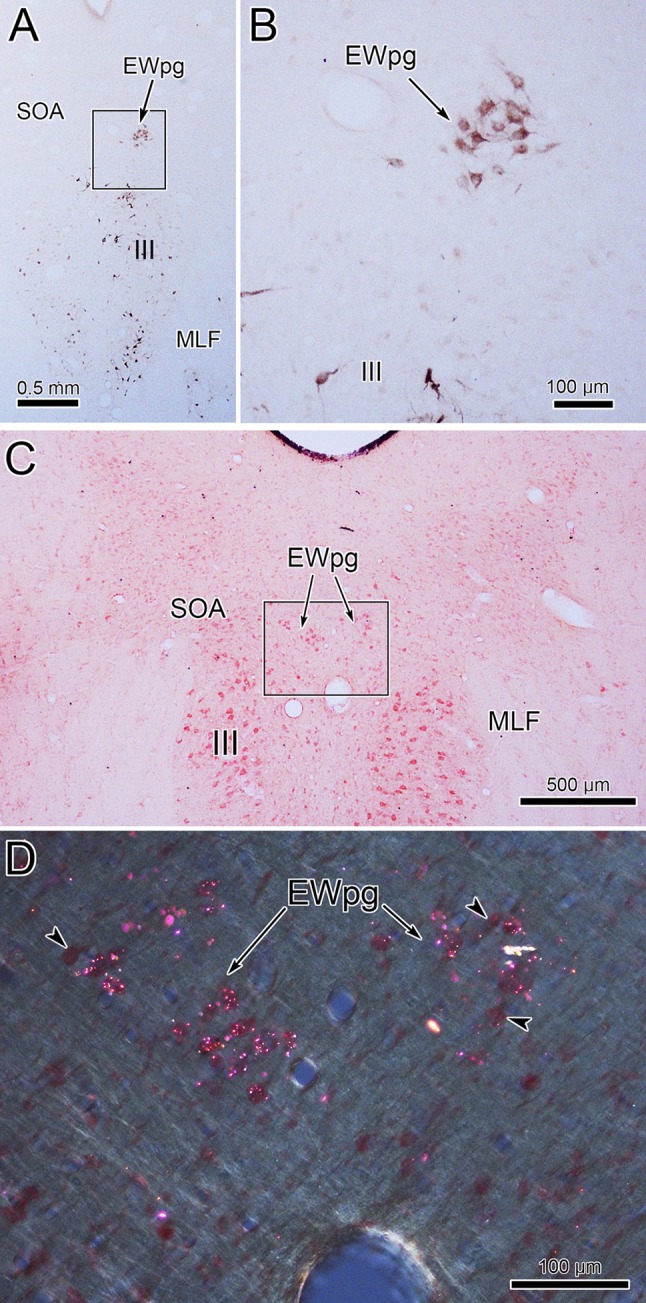
Trans-synaptic labeling of preganglionic motoneurons. **a**, **b** Trans-synaptic retrograde labeling following injection of WGA into the vitreous chamber. The lower magnification view (**a**) reveals the presence of some motoneuron labeling in III. Box indicates region shown at higher magnification in (**b**). Gray/black, trans-synaptic labeling was quite extensive within the EWpg. **c**, **d** Trans-synaptic retrograde labeling following injection of WGA–HRP into the vitreous chamber (right) and anterior chamber (left). A box in the bright-field view (**c**) indicates the area demonstrated at higher magnification with crossed polarizer illumination in (**d**). Most of the motoneurons in EWpg on both sides showed small bright crystals of reaction product. Some cells in EWpg lacked label (arrowheads), but none of the cells in III showed label (not illustrated)

**Fig. 4:**
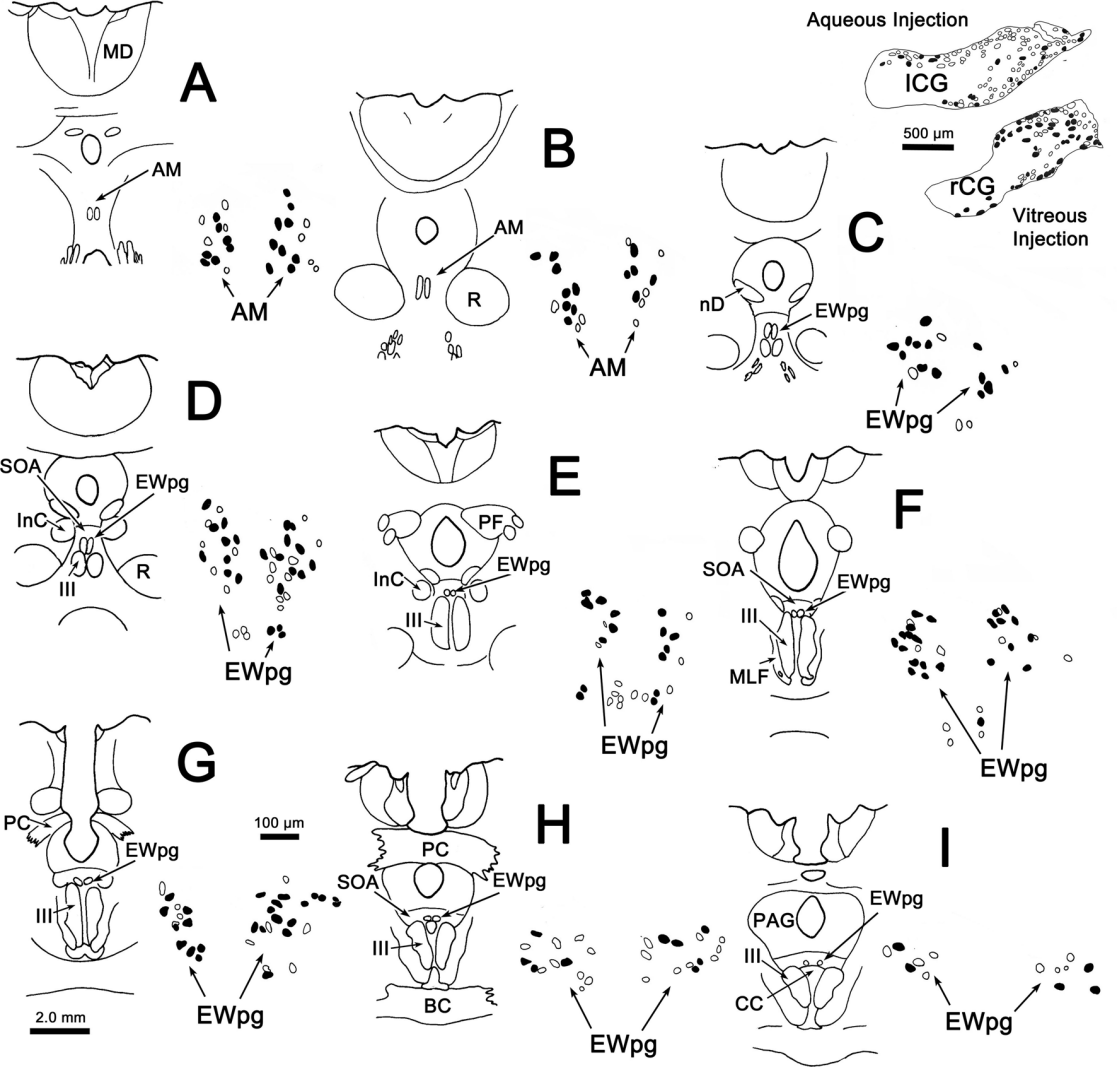
Distribution of trans-synaptically labeled preganglionic motoneurons. Inserts in the upper right show the pattern of WGA–HRP-labeled (filled) and -unlabeled (empty) postganglionic motoneurons in the ciliary ganglia. Fewer cells were labeled from the left aqueous injection than the right vitreous injection. A rostral (**a**) to caudal (**i**) series of sections illustrates the AM and EWpg, with low (left) and high (right) magnification views. Arrows indicate the left and right AM and EWpg nuclei in the high-magnification view, where trans-synaptically labeled (filled profiles) preganglionic motoneurons outnumber unlabeled motoneurons (empty profiles). Roughly equal numbers of labeled cells were present on the two sides

In an attempt to produce labeling of preganglionic motoneurons without attendant labeling of extraocular motoneurons, we utilized trans-synaptic transport of WGA injected into the anterior or vitreous chambers of the eye. Figure 3a, b shows motoneurons in EWpg that were labeled trans-synaptically with WGA and revealed immunohistochemically. The labeled cells contained numerous black punctate chromogen granules that gave the cells a gray/black tone. However, we still observed scattered extraocular motoneuron labeling within III, indicating WGA spreads outside the globe. We then employed WGA–HRP injections, reasoning that a larger molecule would be less likely to escape the confines of the globe. Trans-synaptic label was only observed in one of four cases injected. Figure 3c, d shows an example in which trans-synaptically transported WGA–HRP was observed. In this case, crossed polarizers revealed the fine speckling of the labeled cells with the reaction product. This labeling was not present in extraocular motoneurons. The distribution of the labeled preganglionic motoneurons in this case is further demonstrated in the chartings of Fig. 4. This animal received an aqueous injection of WGA–HRP into the anterior chamber of the left eye and a vitreous injection of WGA–HRP into the right eye. The left ciliary ganglion contained fewer retrogradely labeled motoneurons (filled profiles) than the right ciliary ganglion. However, the number of labeled cells from the anterior chamber injection indicates that we failed to specifically label the pupillary population, which is believed to be under 10% (Warwick 1954). At the level of the EWpg, the number of trans-synaptically labeled cells (filled profiles in Fig. 4a–i) is approximately the same on the two sides, suggesting the degree of divergence in the preganglionic projection is such that numerous preganglionic motoneurons can be trans-synaptically labeled from a few labeled postganglionic motoneurons. The organization of each labeled cell column is remarkably similar to that seen following the ganglion injection (Figs. 1 and 2). At rostral levels, the labeled cells occupied AM (Fig. 4a, b). At the level of III, the labeled cells were mainly found in paired nuclei within SOA (Fig. 4c–g). Once the caudal central subdivision appears, the preganglionic motoneurons soon end (Fig. 4h, i). In some places, the distribution of the labeled EWpg cells was less compactly organized (Fig. 4d, e).

### Pretectal projections

**Fig. 5:**
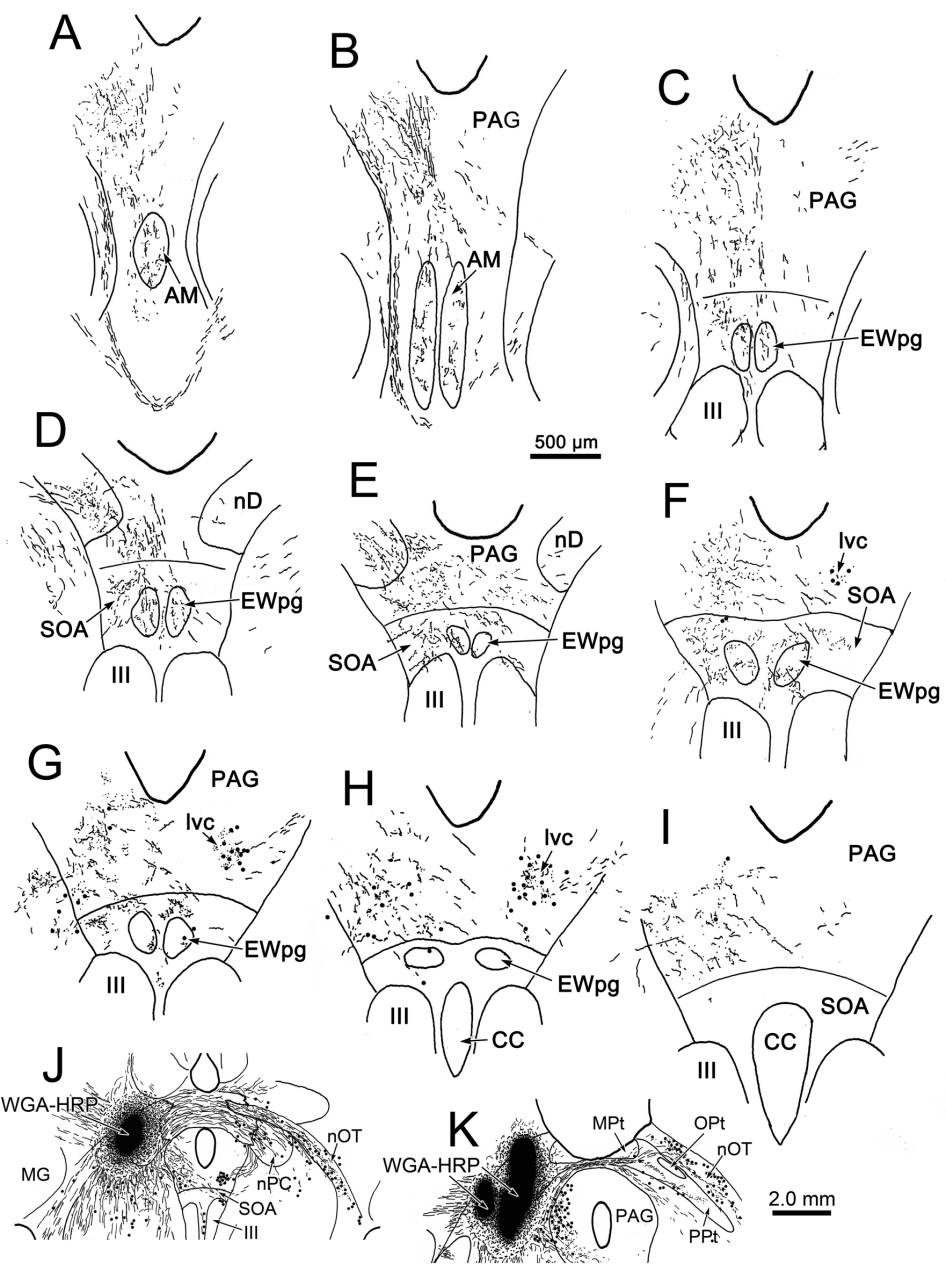
Pattern of labeling observed following a pretectal injection of WGA–HRP. The injection site in the pretectum (**j**, **k**) includes the OPt, plus the surrounding nOT and PPt. Labeled axons (lines), terminals (stipple) and cells (dots) located beneath the cerebral aqueduct are illustrated in a rostral (**a**) to caudal (**i**) series. Note the presence of terminals in AM, PAG, SOA and EWpg, and cells in the contralateral lvc

**Fig. 6:**
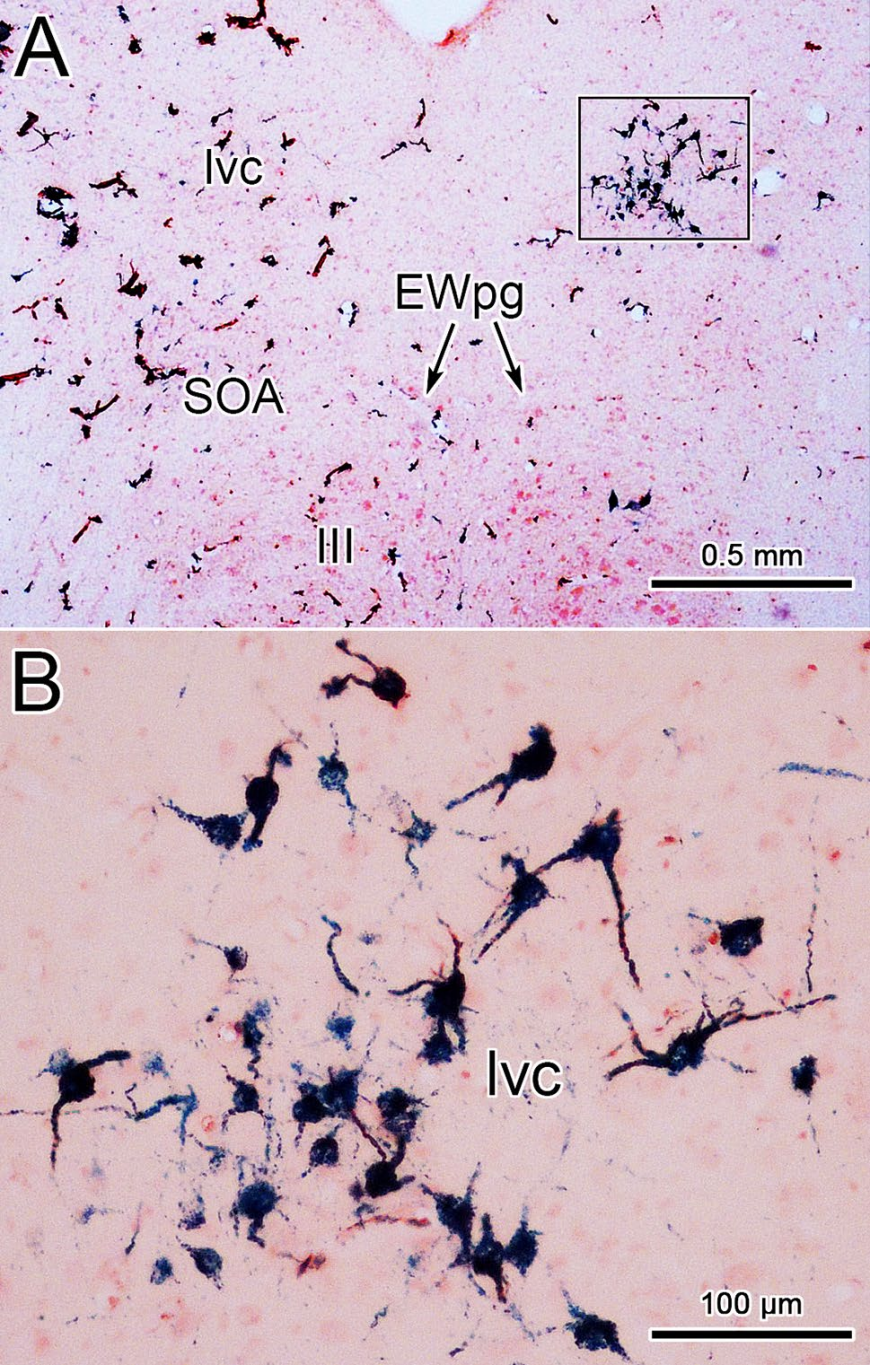
Labeled neurons in the lateral visceral column (lvc). Lower (**a**) and higher (**b**) magnification views of labeled cells from the case illustrated in Fig. 5. Only the nucleus contralateral to the WGA–HRP injection site contains labeled multipolar cells with small spherical somata. Box in (**a**) indicates area shown in (**b**)

Figure 5j, k shows a large injection of WGA–HRP that encompassed much of the pretectum, including the OPt. Note the large number of axons decussating in the posterior commissure. The resultant pattern of anterogradely labeled axons (lines) and terminals (stipple) beneath the aqueduct is illustrated (Fig. 5a–i). Rostral to III, terminations were present within AM (Fig. 5a, b). At the level of III (Fig. 5c–h), numerous terminations were present in the SOA and within EWpg. Note that these terminations were bilateral, but slightly more were found ipsilaterally. In some cases, labeled axons could be observed crossing the midline beneath the aqueduct (Fig. 5d–h), suggesting that the posterior commissure is not the only route by which pretectal axons access the contralateral side. The distribution of terminations was not homogeneous along the rostrocaudal axis of EWpg; more were found in the middle of the nucleus (Fig. 5d–f) and relatively few were observed caudally (Fig. 5g–i). Note that considerable terminal labeling was also present in the periaqueductal gray (PAG) dorsal to the SOA on the ipsilateral side (Fig. 5e–i). The WGA–HRP also retrogradely labeled neurons (dots). These were numerous and well organized in a region of the PAG just dorsal to the SOA on the contralateral side (Fig. 5f–h). We have termed the region containing these labeled pretectal efferents, the lateral visceral column (lvc). The location and appearance of the retrogradely labeled neurons within the lvc is shown in Fig. 6a, b. These lvc cells have relatively small somata compared to the preganglionic motoneurons.

**Fig. 7:**
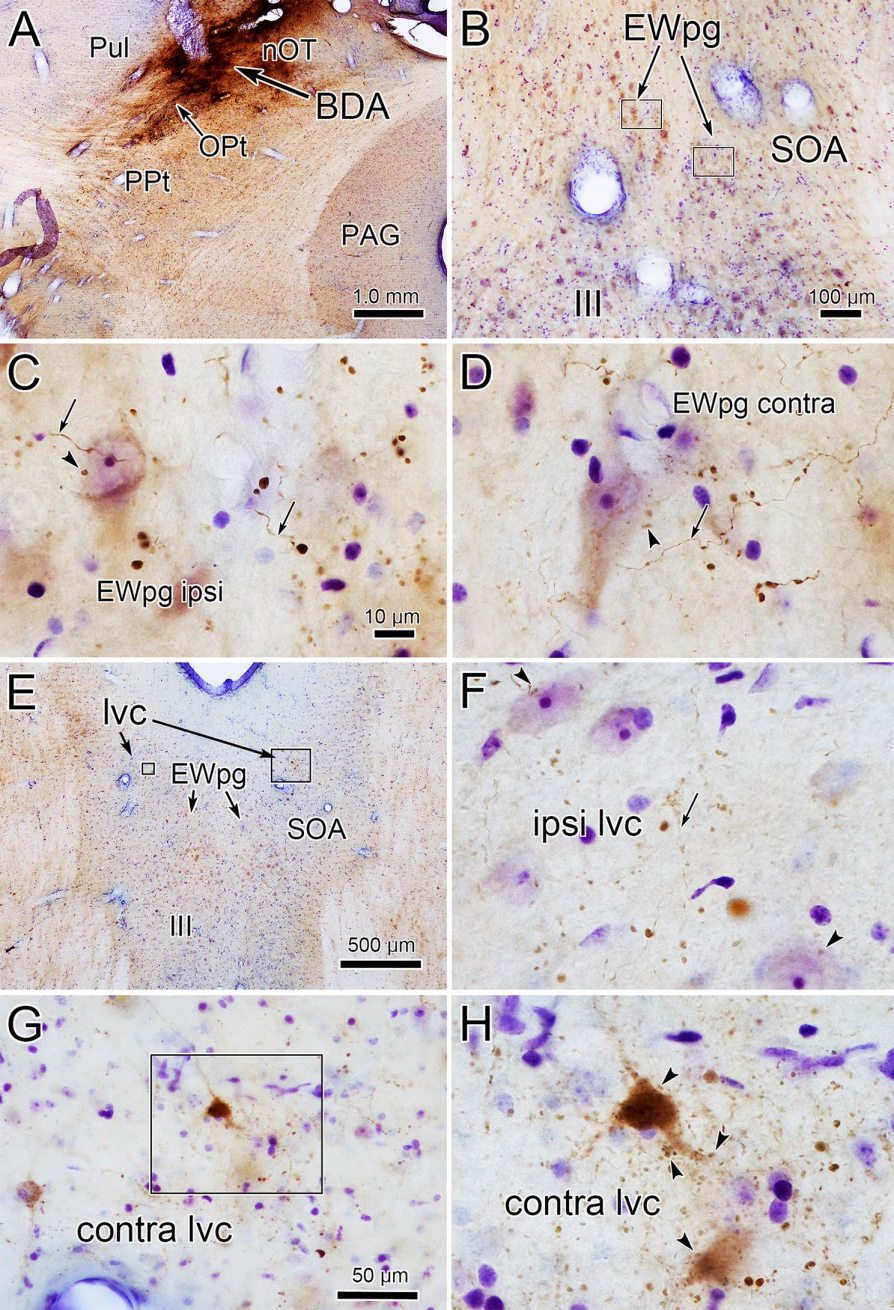
Morphology of anterogradely labeled pretectal axons. The BDA injection shown in (**a**) included the left OPt and spread into the adjacent nOT and Pul. Within the SOA shown in (**b**), labeled axons (arrows) and boutons were present in both the ipsilateral (ipsi) (**c**) and contralateral (contra) (**d**) EWpg, and displayed close associations (arrowheads) with large counterstained neurons. **e**–**h** Labeling in the lvc. The area in the small box in (**e**) is shown in (**f**) and in the large box is shown in (**g**). The boxed area in (**g**) is shown at even higher magnification in (**h**). Labeled axons (arrow) and terminal fields were present in the lvc ipsilateral (**f**) and contralateral (**g**, **h**) to the injection site. On the ipsilateral side (**f**), close associations (arrowheads) with counterstained somata are present. On the contralateral side (**h**), close associations (arrowheads) with retrogradely labeled neurons are present. Scale in c = d, f, h

**Fig. 8:**
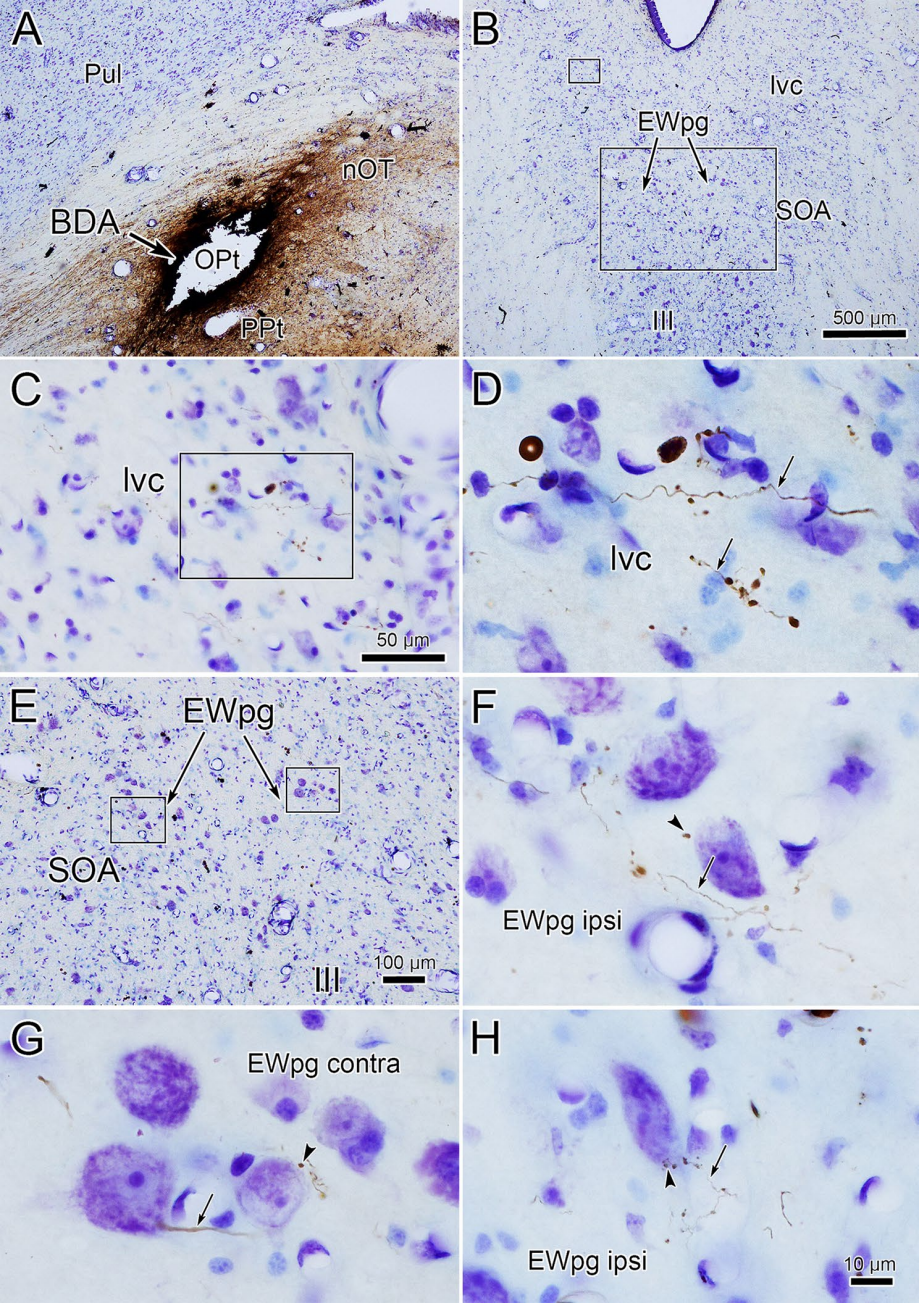
OPt axons in the lvc and EWpg. **a** A small OPt injection of BDA had only slight spread into the PPt and nOT. **b** Two regions are illustrated—the area in the small box located in the lvc is shown in (**c**) and the area in the large box containing EWpg is shown in (**e**). The labeled axons (arrows) in the lvc ipsilateral to the injection site contained in the boxed area in **c** are shown at higher magnification in (**d**). Samples from the EWpg ipsilateral (ipsi) and contralateral (contra) to the injection site (boxes in **e**) are shown in (**f**) and (**g**), respectively. An ipsilateral EWpg example from another section is shown in (**h**). The labeled OPt axons (arrows) display numerous en passant boutons, some of which form close associations with large counterstained somata (arrowheads). Scale in **b** = **a**, **h** = **d**, **f**, **g**

Since the WGA–HRP injection of the pretectum was relatively large, we examined the pattern of anterograde labeling observed following smaller BDA injections. One such injection that included the OPt is shown in Fig. 7a. This injection site also included portions of the nucleus of the optic tract (nOT) and nucleus of the posterior commissure, as well as the pulvinar. BDA-labeled axons were common throughout the SOA, and labeled boutons were observed in close association (arrowheads) with neurons within the ipsilateral (Fig. 7c) and contralateral (Fig. 7d) EWpg. In addition, numerous anterogradely labeled terminal arbors were present in the ipsilateral lvc (Fig. 7f), and some were closely associated with counterstained somata (arrowhead). Anterogradely labeled terminals were also observed contralaterally in lvc, and some were in close association (arrowheads) with the retrogradely labeled cells, suggesting synaptic contact (Fig. 7g, h). A smaller BDA injection of the OPt also labeled terminals at these sites (Fig. 8a). This injection only spread slightly into the posterior pretectal nucleus (PPt) and nOT. BDA-labeled terminal arbors were observed in the ipsilateral lvc (Fig. 8e, f) and in the ipsilateral (Fig. 8f, h) and contralateral (Fig. 8g) EWpg, where labeled boutons were observed in close association (arrowheads) with the larger counterstained somata. The two pretectal PhaL injections did not include the OPt and so acted as controls. One involved nOT and the other PPt. They produced only a few labeled terminals in EWpg.

### Dual-tracer labeling

**Fig. 9:**
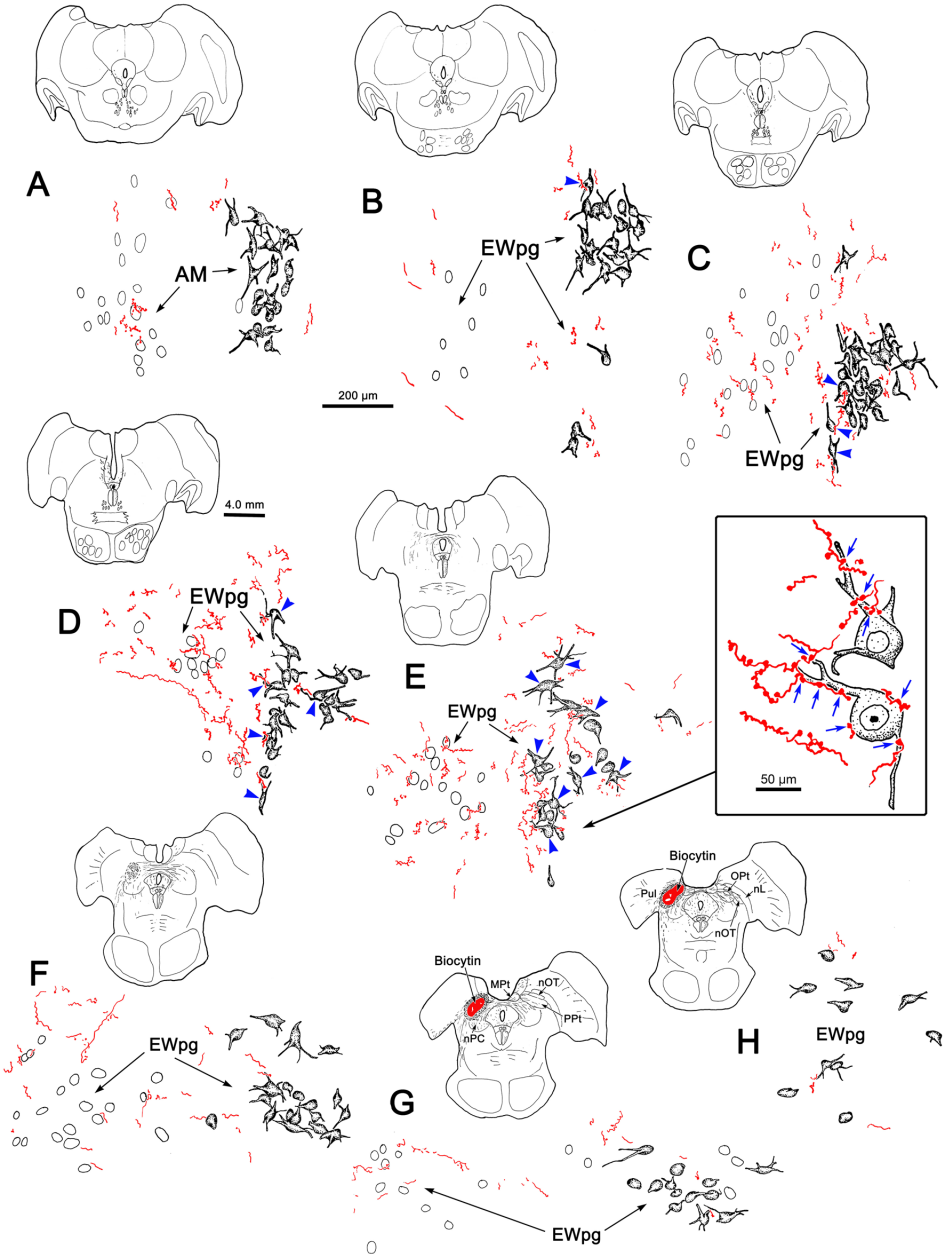
Distribution of pretectal axons with respect to preganglionic motoneurons in the Edinger–Westphal nucleus. **a**–**h** Low-magnification illustration shows the sections in a rostral to caudal series, and the high-magnification illustrations show the region of AM and EWpg for each section. The retrogradely labeled motoneurons from a ciliary ganglion injection of WGA–HRP are shown on the right and counterstained somata of the nuclei are shown on the left. An injection of biocytin (red shading) in the pretectum (**g**, **h**) that included OPt anterogradely labeled axons (red) in EWpg on both sides. The boxed region shows a higher magnification view of two cells in (**e**), which displayed numerous close associations (blue arrows) with BDA-labeled pretectal axons. Labeled cells that showed close associations are indicated by blue arrowheads (**c**–**e**). NB: the dispersed nature of EWpg in (**h**) made it difficult to define the nucleus on the counterstained side

**Fig. 10:**
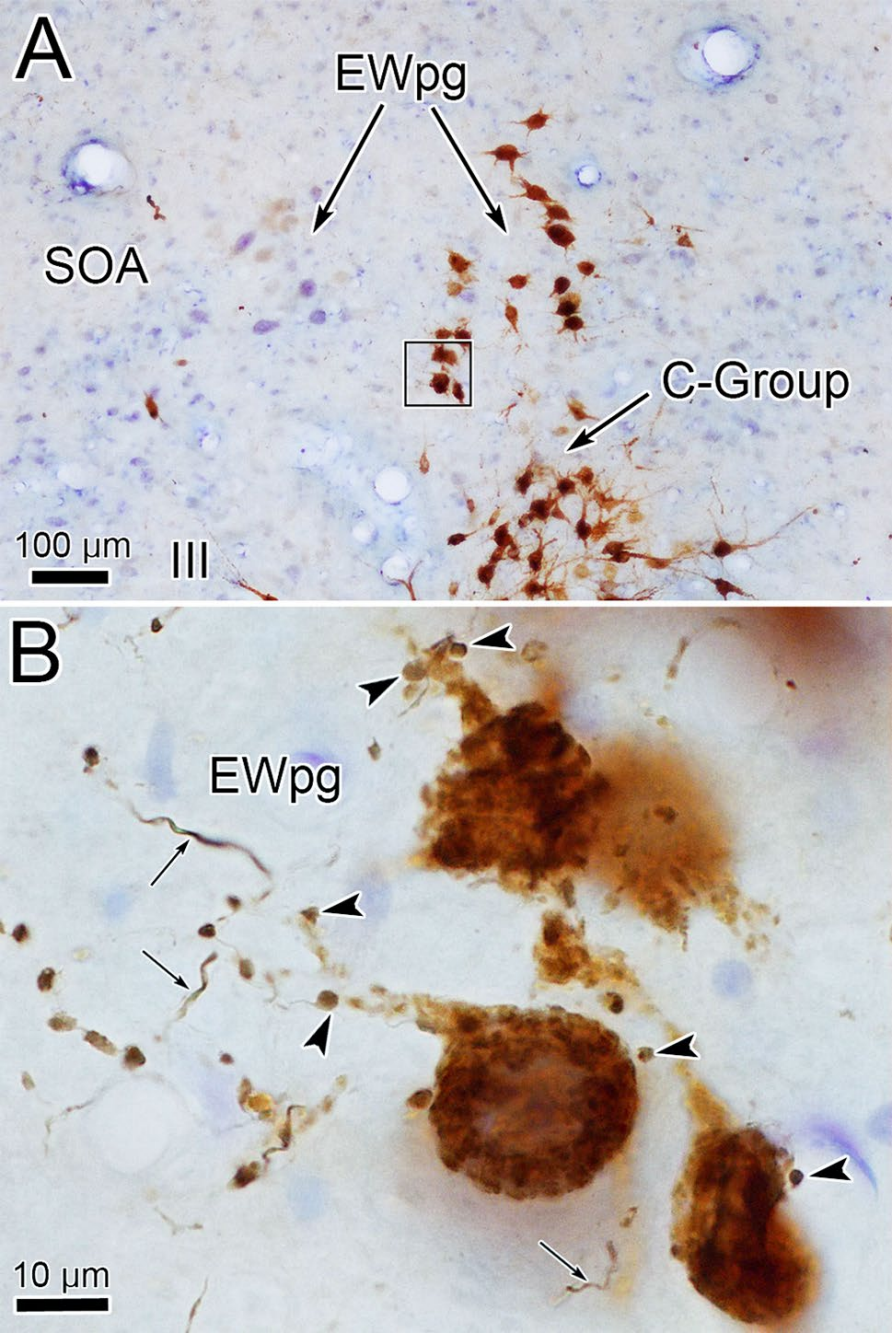
Pretectal input to preganglionic motoneurons. **a** Low-magnification view of the region containing brown, retrogradely labeled motoneurons in EWpg, C-group and the III. Boxed region shown at higher magnification in (**b**). Labeled axons (arrows) arborize and display numerous boutons, some of which display close associations (arrowheads) with the labeled preganglionic motoneurons

We utilized a dual-tracer experiment to confirm whether pretectal terminals directly contact preganglionic motoneurons. In the illustrated case, a biocytin injection fills much of the left pretectum (Fig. 9g, h, section drawings). The resultant anterogradely labeled axons were distributed throughout the ipsilateral and contralateral EWpg (Fig. 9b–h) and in the area of AM containing preganglionic motoneurons (Fig. 9a). Motoneurons retrogradely labeled from the ciliary ganglion injection fill the entire nuclear column on the right side. Labeled cells displaying close associations with labeled boutons are indicated by arrowheads. These are most common at middle levels of EWpg (Fig. 9c–e). Two cells with numerous contacts located in E are shown at higher magnification in the box. Cells like these, with numerous contacts, were relatively few in number and tended to be located ventrally at mid-rostrocaudal levels of EWpg. Figure 10a, b shows the appearance of this population. Several close associations (arrowheads) are evident between biocytin-labeled pretectal terminals and WGA–HRP-labeled preganglionic motoneurons contralateral to the pretectal injection site. Note that both en passant and terminal boutons were present in the immediate vicinity of the retrogradely labeled motoneurons.

### Ultrastructure of preganglionic motoneurons and inputs

**Fig. 11:**
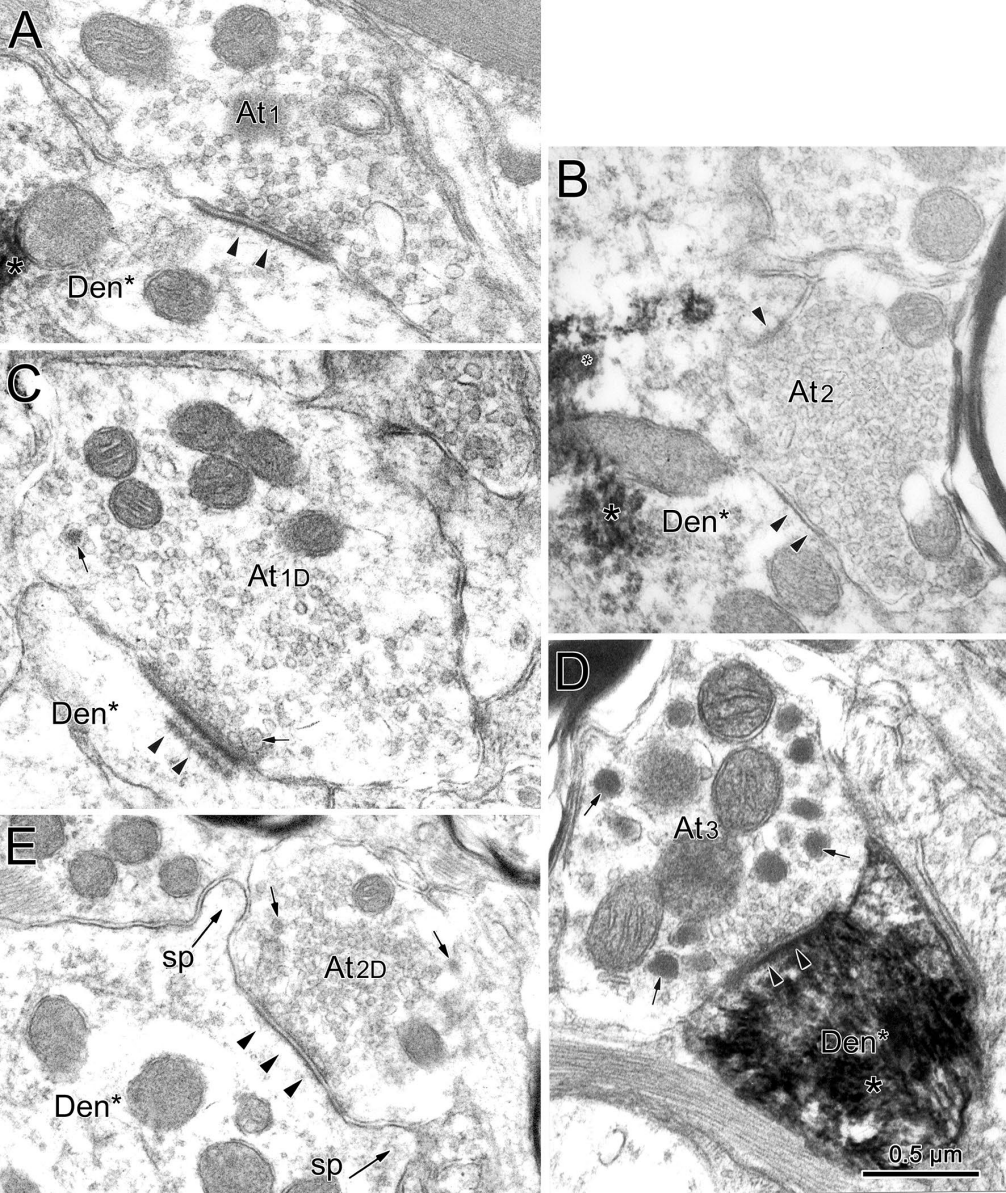
Terminal types contacting (arrowheads) preganglionic motoneurons. Chromogen (asterisk) in the motoneurons labeled from the ciliary ganglion had an electron-dense, fibrous appearance. Terminal profiles were categorized as type At1 if they contained clear spherical vesicles (**a**), type At2 if they contained clear pleomorphic vesicles (**b**), type At1D if they contained clear spherical vesicles plus scattered dense-core vesicles (small arrows) (**c**), type At2D if they contained clear pleomorphic vesicles plus scattered dense-core vesicles (**e**), and type At3 if large dense-core vesicles were the dominant feature (**d**). Generally, At1 and At1D type terminals had asymmetric synaptic contacts, although the extent of the postsynaptic density in the synapse (arrowheads) varied (**a**, **c**), while At2 and At2D type terminals had symmetric synaptic contacts (**b**, **e**). In some asymmetric examples, terminal bars were present (**c**). Scale bar in **d** = **a**–**c**, **e**

**Fig. 12:**
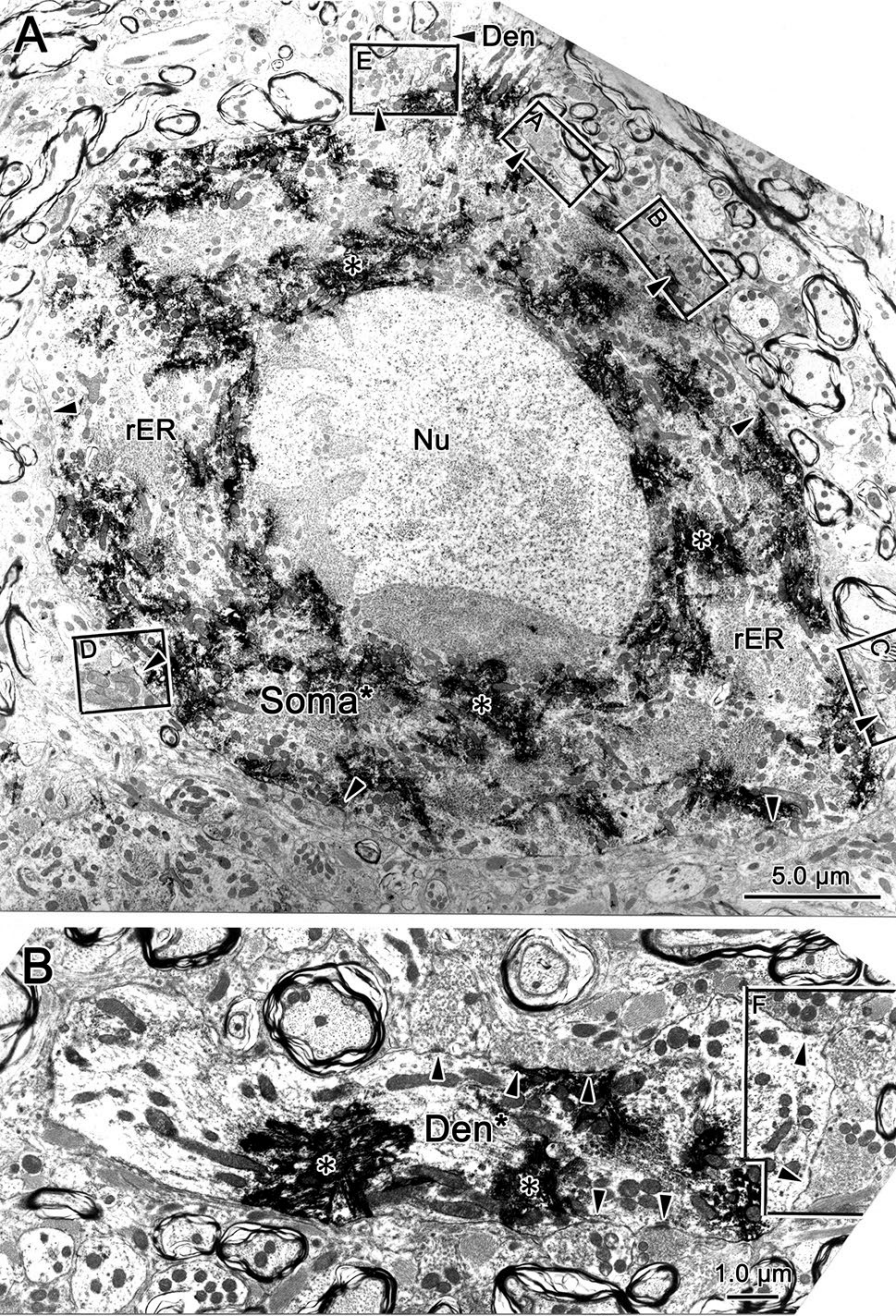
Retrogradely labeled (asterisk) preganglionic motoneuron soma and dendrite from the EWpg. **a** The soma displays prominent stacks of rough endoplasmic reticulum. Synaptic contacts (arrowheads) are scattered along its surface. **b** The longitudinally sectioned dendrite shows terminals clustered over areas of its surface. Selected contacts (lettered boxes) are shown at higher magnification in Fig. 13

Retrogradely labeled neuronal profiles could be discerned by the presence of electron-dense, fibrous-appearing inclusions (asterisk) within the cytoplasm of dendrites (Figs. 11a, b, d; 12b) and somata (Fig. 12a). Often these appeared as just a patch; but in some cases, they filled the dendritic profile (Fig. 11d). The extents of the observed pre- and postsynaptic densities and the number of non-spherical vesicles in a profile varied among the profiles. While it might be argued that this variance represents a spectrum, for the purposes of classification, we nevertheless attempted to divide these two profile characteristics in a binary fashion and place the terminals contacting labeled cells into one of the five categories. Type 1 (At1) profiles contained clear, mostly spherical vesicles and often displayed asymmetric synaptic densities in which the postsynaptic element predominated (Fig. 11a). Type 2 (At2) profiles contained clear vesicles having a variety of shapes and displayed symmetric synaptic densities in which the pre- and postsynaptic elements were approximately equal in predominance (Fig. 11b). In some cases, a few small dense-core vesicles were present as well. When these dense-core vesicles were observed in a terminal containing spherical vesicles, we classified it as a Type 1D profile (At1D) (Fig. 11c). When these dense-core vesicles were observed in a terminal containing pleomorphic vesicles, we classified it as a Type 2D profile (At2D) (Fig. 11e). The fifth type of profile was very rare. It contained numerous large dense-core vesicles, along with a small number of clear pleomorphic vesicles. We classified these profiles as Type 3 (At3) (Fig. 11d).

**Fig. 13:**
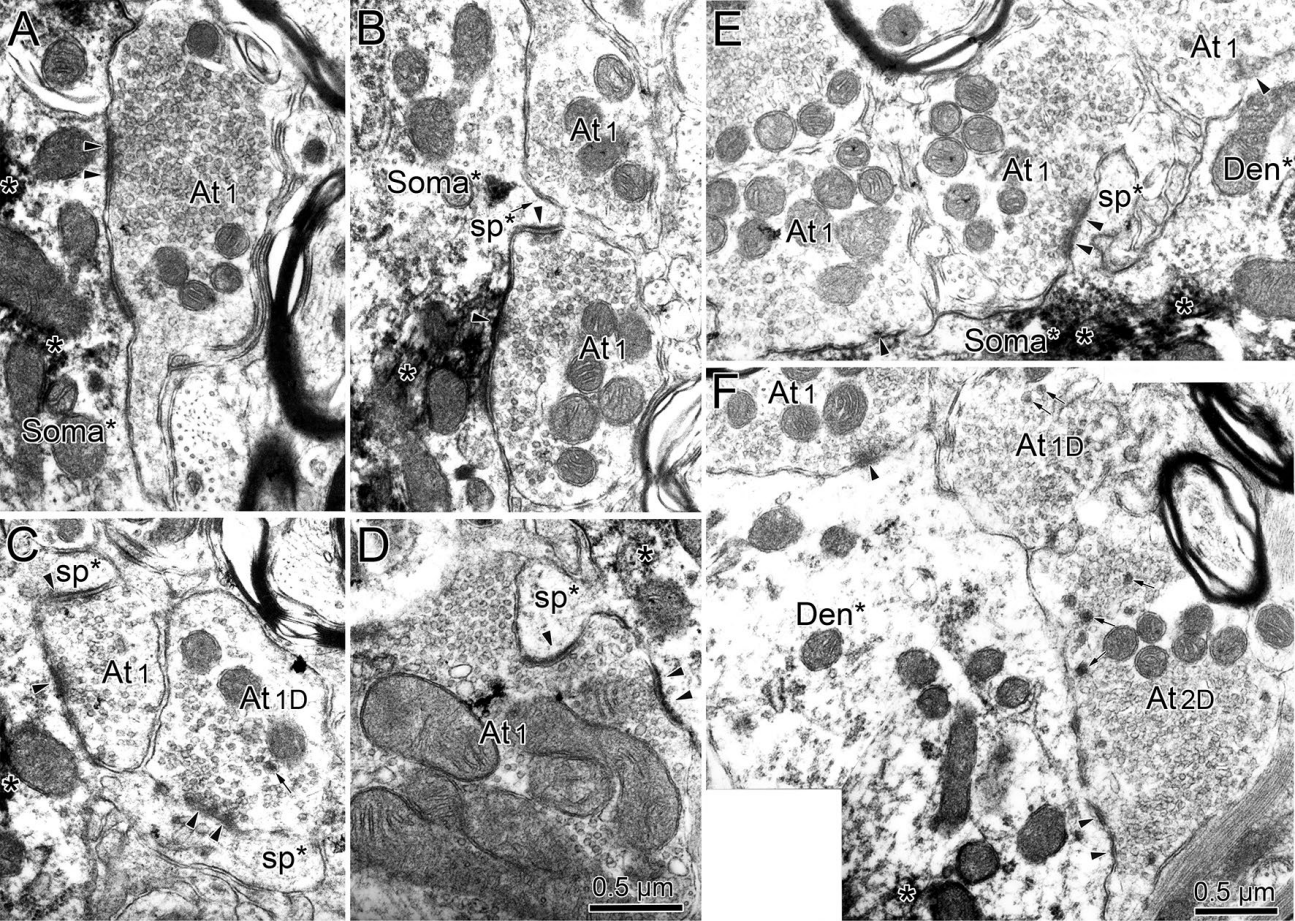
Axosomatic (**a**–**e**) and axodendritic (**f**) synaptic profiles contacting labeled (asterisk) preganglionic motoneurons shown in Fig. 12. Numerous synaptic contacts (arrowheads) were observed on the plasma membrane of the soma (**a**–**e**). In addition, the somata often displayed spines of various shapes and lengths (**b**–**e**) that were synaptically contacted by terminals. Most axosomatic profiles contained clear spherical vesicles (At1), although some contained scattered dense-core vesicles (small arrow), as well (At1D) (**c**). At1 and At1D type profiles also predominated on the proximal dendrites (**f**), but profiles with clear pleomorphic vesicles (At2D) were also observed. Scale bar in **d** = **a**–**c**), **e**

Typical examples of a soma and a large dendrite belonging to a retrogradely labeled preganglionic motoneuron are shown in Fig. 12. The nucleus of the labeled soma (Soma*) is euchromatic and indented (Fig. 12a). The cytoplasm features large stacks of rough endoplasmic reticulum and many mitochondria. These cytoplasmic features are also present in labeled, large-diameter proximal dendrites (Den*, Fig. 12b). The plasma membrane of the soma is covered by a number of terminal profiles that make synaptic contact (arrowheads, Fig. 12a). The plasma membrane of the proximal dendrite is similarly contacted (arrowheads, Fig. 12b). In both cases, the terminals are often clustered together, so a considerable portion of the plasma membrane is free of synaptic profiles. Some of the examples of these contacts are indicated by boxes and are shown at higher magnification in Fig. 13. All of the examples of axosomatic contacts shown here are Type 1 profiles that contained numerous clear spherical vesicles (Fig. 13a–d), and this type of contact was quite common on somata. Note that the asymmetric nature of the synaptic density is not always evident. These cells often displayed spinous processes of various shapes (sp. Fig. 13b–e). The higher magnification views of three contacts on the example of a proximal dendrite shown in Fig. 12 reveal that they also contained clear spherical vesicles. However, two of these examples also contained scattered dense-core vesicles, and so were classified as Type 1D terminals (Fig. 13f). Type 1 and 1D contacts were quite common on proximal dendrites.

**Fig. 14:**
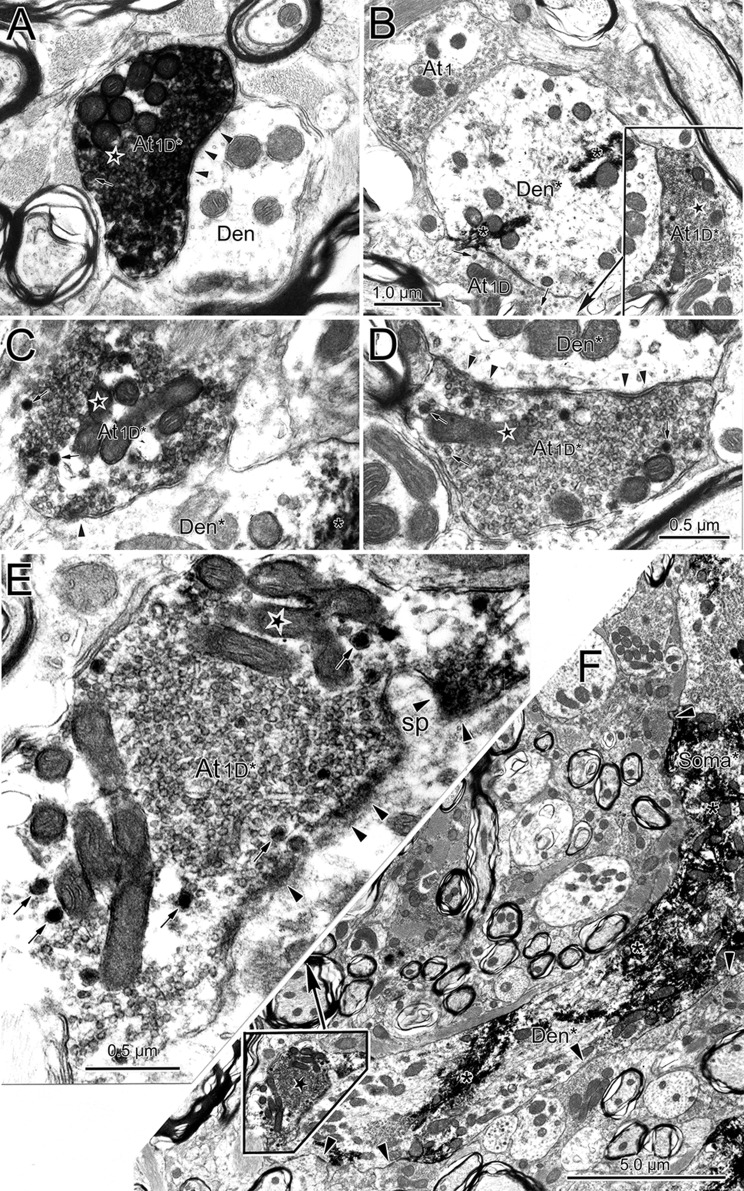
Monosynaptic pretectal input onto preganglionic motoneurons. **a** An example of a very electron-dense, anterogradely labeled (star) pretectal terminal (At1D*) synapsing (arrowhead) on an unlabeled dendrite. **b** A retrogradely labeled (asterisk) preganglionic motoneuron dendrite is shown in cross section. Three terminal profiles lie adjacent to it. Two are unlabeled (At1 and At1D). A third, labeled profile is enclosed in a box and shown at higher magnification (**d**). It contains numerous clear spherical vesicles and a few dense-core vesicles (arrows), so it is classified at At1D. Another example with pre- and postsynaptic labeling is shown (**c**). The heavily labeled preganglionic motoneuron soma and proximal dendrite shown at low magnification (**f**) is contacted by an anterogradely labeled terminal. The boxed area containing this terminal is shown at higher magnification (**e**). Reaction product produces an electron-dense coat around the synaptic vesicles. This profile is classified as At1D. It makes asymmetric synaptic contacts (arrowheads) with both the dendritic shaft and a small spine. Scale bar in **d** = **a**, **c**

Next, we examined the ultrastructure and organization of pretectal terminals within the EWpg. A biocytin injection into the pretectum that included the OPt (Fig. 9g, h) produced anterogradely labeled terminals (At*) like those shown in Fig. 14. The reaction product appeared as an electron-dense fuzz around the vesicles in the profile. In some cases, the labeling was so dense that it was difficult to discern the terminal contents (Fig. 14a). In other cases, the labeling was more subtle (Fig. 14b–f), but labeled terminals could still be distinguished from unlabeled ones that had a more electron-lucent appearance (At1 and At1D, Fig. 14b). The anterogradely labeled profiles made asymmetric synaptic contacts (Fig. 14a) and contained numerous clear spherical vesicles and scattered dense-core vesicles, indicating they are Type 1D terminals (At1D*; Fig. 14a, c–e). While many of the terminals contacted profiles in which reaction product was not evident (Den, Fig. 14a), examples where retrograde tracer was observable were also found (Den*, Fig. 14b–f). This indicates that a monosynaptic input from the pretectum onto preganglionic motoneurons in EWpg is present.

### Quantitative analysis

**Fig. 15:**
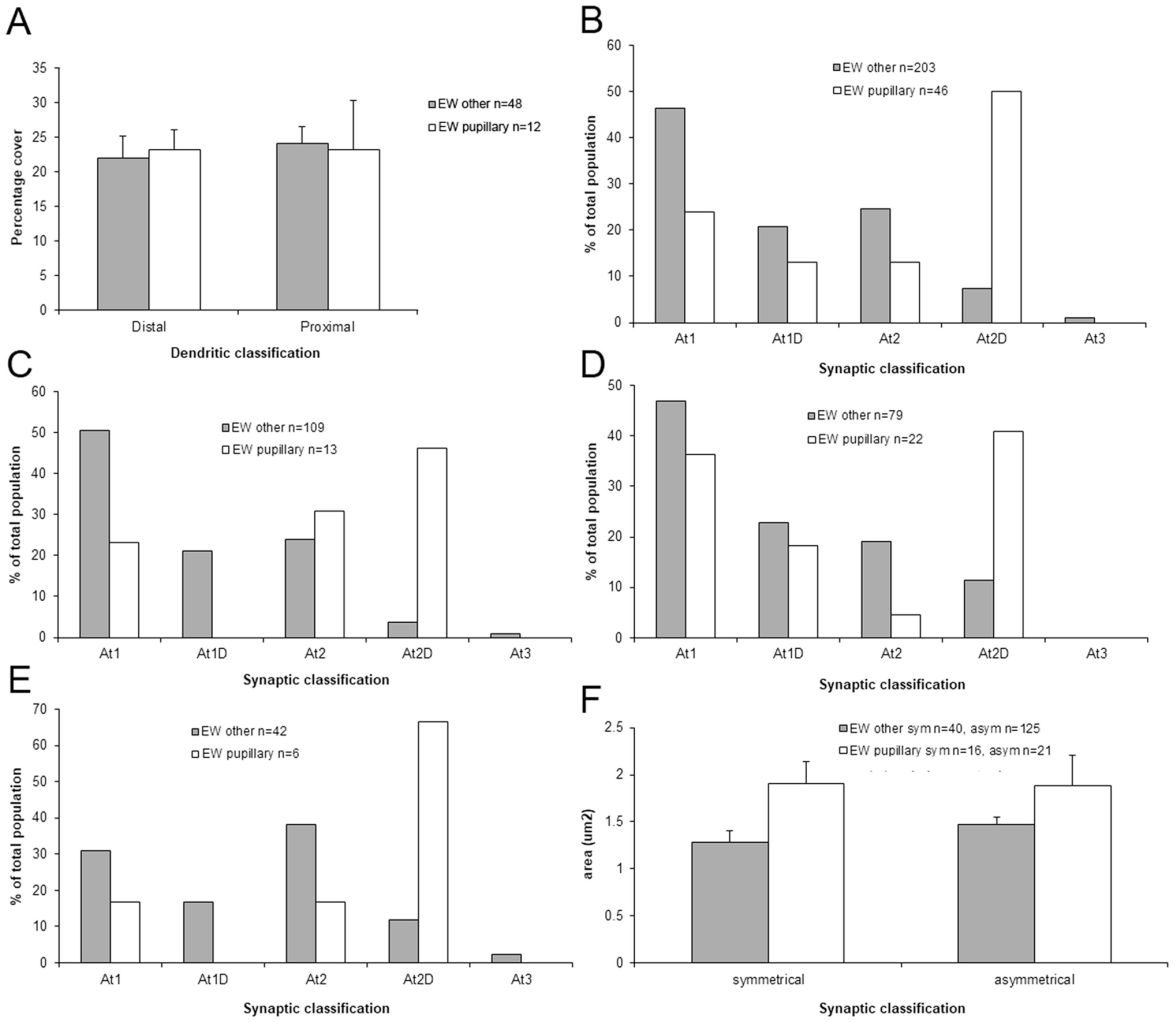
Quantitative comparisons of the ultrastructural characteristics of preganglionic motoneurons. Profiles receiving pretectal input are termed ‘pupillary’ and those not observed receiving input are termed ‘other’. **a** Comparison of membrane coverage of distal and proximal dendrites. **b** Comparison of the percentage of terminals contacting preganglionic motoneurons broken down by terminal type. **c**–**e** Comparison of the percentage of terminal contacting labeled somata (**c**), proximal dendrites (**d**) and distal dendrites (**e**) broken down by terminal type. **f** Comparison of sizes of terminals which displayed symmetric or asymmetric synaptic densities with respect to the two preganglionic motoneuron populations

We quantified a number of aspects of the ultrastructure of the retrogradely labeled preganglionic motoneurons. The means of the basic dimensions of the labeled somata and dendrites are found in Table 2. When these retrogradely labeled motoneuron examples were divided into those displaying inputs from labeled pretectal terminals (*n* = 45), termed pupillary motoneurons, and those that did not display labeled pretectal inputs (*n* = 200), termed other motoneurons, there was no statistical difference in any of these measures of motoneuron characteristics. Of course, it should be noted that the “other” category would include a minority of pupillary motoneuron profiles in which a labeled pretectal contact was not evident, amongst the majority of lens-related motoneuron profiles. We also compared the degree of synaptic coverage of dendrites with respect to the pupillary and other category of motoneurons (Fig. 15a). Little difference was seen in terms of coverage of either the proximal or distal dendrites.

**Table 2:** Quantitative ultrastructural findings

Profile	Perimeter	Major axis	Minor axis	Profile contact	Synaptic contact
Somata *n* = 13	102.23 ± 8.86	30.25 ± 1.97	19.85 ± 1.26	15.12 ± 2.41	3.30 ± 0.42
All dendrites *n* = 47	37.43 ± 5.20	11.76 ± 1.51	7.00 ± 1.03	7.12 ± 0.93	1.52 ± 0.19
Proximal dendrites *n* = 33	47.27 ± 6.26	14.90 ± 1.80	8.73 ± 1.21	7.95 ± 1.20	1.69 ± 0.23
Distal dendrites *n* = 14	9.16 ± 1.29	3.16 ± 0.48	1.66 ± 0.16	1.94 ± 0.30	0.45 ± 0.08

Mean measures and standard errors in micrometer for retrogradely labeled profiles in EWpg

We then turned our attention to the types of terminal in synaptic contact with the retrogradely labeled preganglionic motoneurons. The axon terminals (*n* = 226) innervating these motoneurons showed a majority (*n* = 103) of Type 1 profiles, with fewer Type 2 (*n* = 60) and Type 1D (*n* = 45) terminals. There were relatively few Type 2D terminals (*n* = 18), and Type 3 terminals (*n* = 3) were very rare. In this case, we did see a clear difference between the pupillary and other preganglionic motoneuron samples (Fig. 15b). The axon terminals (*n* = 55) contacting pupillary motoneurons showed a majority (*n* = 26) of Type 2D profile type, in which the vesicles were mostly clear pleomorphic with some scattered dense-core vesicles. In fact, this type was 39.2% more common on pupillary motoneurons than on other motoneurons. Type 1 profiles were relatively less common (*n* = 15), and equal quantities of clear Type 1D (*n* = 7) and Type 2 (*n* = 7) were present. No Type 3 profiles were observed on pupillary motoneurons. When we broke this analysis down with respect to neuronal geography, the preponderance of At2D profiles on pupillary neurons was still evident on the membranes of somata (Fig. 15c), proximal dendrites (Fig. 15 d) and distal dendrites (Fig. 15e). It is noteworthy that the terminal type associated with the actual pretectal input in the pupillary population of motoneurons, Type 1D, was only sampled on pupillary proximal dendrites (Fig. 15c–e). However, we found examples in which proximal dendrites that were contacted by labeled pretectal terminals displayed continuity with distal dendrites or somata (Fig. 14e, f).We analyzed these examples to characterize more of the pupillary population. Also of note is the fact that Type A1 terminals appear to be much less common on the distal dendrites of both motoneuron types, compared to the proximal dendrites and somata (Fig. 15c–e).

Several characteristics of the four main terminal types were measured. The mean terminal area ranged between 1 and 2 µm^2^, and varied slightly between terminal classes, but no significant differences were found between the areas of the terminal classes that contact the two motoneuron categories. Similarly, the mean length of the synaptic contacts ranged between 0.35 and 0.55 µm for these four types of profile, but the profile lengths of these different classifications revealed no significant differences for the two types of motoneuron.

Since we were struck by the unusual prevalence of the relatively rare Type 2D terminal on the pupillary preganglionic motoneurons, we undertook a separate comparison using just synaptic contact characteristics. We confined this analysis to samples where we were confident of the symmetrical or asymmetrical classification. We found no significant difference between the two motoneuron categories with respect to the length of profile apposition, the actual synaptic density length or the synaptic coverage. However, we did see differences with respect to the area of the terminals (Fig. 15f). The mean area of the terminals contacting the pupillary motoneurons was larger than that of terminals contacting motoneurons in the other category. Only the symmetrical terminal measures reached significance (*p* < 0.05). Symmetric contacts on pupillary preganglionic motoneurons had a mean area of 1.90 µm^2^ (s.e. 0.23 µm^2)^, and those contacting the other motoneurons had a mean area of 1.29 µm^2^ (s.e. 0.12 µm^2^).

## Discussion

The results of this study indicate that the motoneurons in the macaque EWpg are organized into a single column that runs longitudinally, dorsal to III. Hopefully, this will lay to rest arguments over whether the monkey EWpg contains cytoarchitectonically distinct subdivisions. The results strongly suggest that the OPt provides a monosynaptic input to a subpopulation of EWpg motoneurons. This is presumably the substrate for the pupillary light reflex, and our data indicate it is a bilateral projection, supporting both the direct and consensual responses. The ultrastructural characteristics of this projection are congruent with the pretectal projection being excitatory, and the presence of dense-core vesicles suggests peptide neuromodulators may be present. Beyond receiving pretectal input, pupillary motoneurons differ from lens-related motoneurons at the ultrastructural level in that they receive a higher percentage of inhibitory inputs. Finally, this study indicates the presence of a feedback projection to the pretectum from a novel source, the lvc. This feedback may play a role in constructing the bilateral retinal fields characteristic of many OPt neurons.

### Technical considerations

Several of the techniques we used to label preganglionic motoneurons also produced labeling of somatic motoneurons. This made it difficult to differentiate extraocular motoneurons found in the C-group from preganglionic motoneurons belonging to EWpg. However, trans-synaptic transport of WGA–HRP, while it only produced light labeling of the preganglionic motoneurons, produced no labeling of somatic motoneurons. The consistency in the labeling pattern in EWpg between these tracer types provides further support for our conclusions.

The small size of the OPt and the fact that it is embedded within other pretectal nuclei made it difficult to inject this nucleus without involving adjacent structures. All of our injections spread to other nuclei, making interpretation of any one case difficult. However, the retrograde tracer studies presented in the companion paper (May and Warren 2019) suggest that there are only a few neurons projecting to the region containing EWpg in the nuclei surrounding OPt. The fact that different tracers produced similar patterns of anterograde labeling in the EWpg and SOA when the OPt was involved strongly supports our claims.

Only one dual-tracer experiment was undertaken. This lessens the impact of the argument in support of the monosynaptic projection of the OPt to the EWpg motoneurons. However, the patterns of label seen in this case were identical to all those observed with single injections of retrograde or anterograde tracer. This consideration also applies to the ultrastructural evidence for this projection. Furthermore, our quantitative analysis comparing pupillary preganglionic motoneurons to other preganglionic motoneurons rests on a relatively small sample where we could observe labeled pretectal terminals contacting labeled motoneurons in EWpg.

### Organization of the Edinger–Westphal nucleus

Although the preganglionic motoneurons of the EW form a discrete nucleus in the birds that have been investigated (Reiner et al. 1983; Gamlin et al. 1984), in most mammals, the cholinergic cells of EWpg do not form a discrete nucleus, and are instead scattered in the vicinity of III (Sugimoto et al. 1978; Toyoshima et al. 1980; Kozicz et al. 2011; Sun and May 2014b). In contrast, the division of the EW that contains peptidergic, centrally projecting neurons (EWcp) often forms a fairly discrete nucleus in most mammals (May et al. 2008a; Kozicz et al. 2011). In primates, on the other hand, the motoneurons of EWpg generally form a more discrete nucleus located dorsal to III and extending into AM (present results; Warwick 1954; Akert et al. 1980; Burde and Loewy 1980; Clarke et al. 1985a; Sun and May 1993; May et al. 2008b), whereas the peptidergic cells of EWcp are somewhat more dispersed, lying on the midline between the oculomotor nuclei, within the SOA and lateral to III (Horn et al. 2008; May et al. 2008a; Kozicz et al. 2011).

It has been suggested that the EWpg of monkeys is divided into a number of cytoarchitectonic subdivisions, including dorsal, lateral and medial visceral columns (Burde 1983; Burde and Williams 1989). However, we believe that there are no distinct cytoarchitectonic subdivisions of EWpg for the following reasons: (1) no subdivisions were observed when an antibody to choline acetyltransferase was used to identify motoneurons dorsal to III (Horn et al. 2008; May et al. 2008a). (2) Subdivisions were not seen using retrograde trans-synaptic transport of rabies virus from the ciliary body (May et al. 2018). (3) In the present study, we did not find subdivisions of EWpg following injections of the ciliary ganglion or through the use of trans-synaptic transport of either WGA or WGA–HRP from the globe.

### Preganglionic motoneuron ultrastructure

The ultrastructural examination of EWpg motoneurons indicated that these cells receive input from five different ultrastructural types of terminal: two presumably excitatory classes containing clear spherical vesicles—At1 and At1D, two presumably inhibitory classes containing clear pleomorphic vesicles—At2 and At2D, and a few terminals dominated by large dense-core vesicles—At3. It is always possible that some of the terminals classified as At1 and At2 might simply represent cuts where the sparse small dense-core vesicles were not present. While this sampling effect may have biased the counts towards the At1 and At2 categories, it seems unlikely that sampling can account for all the terminals present in these two categories, as these were generally more common than the corresponding At1D and At2D classes. Similar terminal types were observed contacting EWpg motoneurons in a cat study although a more complex classification system that also considered vesicle packing was employed (Sun and May 2014b).

The presence of multiple terminal types may reflect the presence of multiple inputs to these cells. For lens-related preganglionic motoneurons, inputs from both the near-response neurons in SOA and disjunctive saccade neurons in the central mesencephalic reticular formation should be present (May et al. 2016, 2018, 2019a). The near triad, which controls the eye with respect to target distance, involves the modulation of vergence angle, lens accommodation and pupillary diameter (Mays 1984; Zhang et al. 1992; McDougal and Gamlin 2015; May et al. 2019b). Consequently, many neurons in both these structures that fire for convergence would be expected to provide both lens-related and pupil-related motoneurons in EWpg with excitatory input (Mays, 1984; Mays et al. 1986; Judge and Cumming 1986; Zhang et al. 1992; Waitzman et al. 2008; Das 2011, 2012). These structures also contain populations that fire for divergent eye movements, so they may also provide EWpg motoneurons with inhibitory inputs. In addition, the pupillary preganglionic motoneurons receive an excitatory input from the OPt for the pupillary light reflex (Gamlin et al. 1995). Here, we have demonstrated that the OPt terminals fall into the At1D category of presumed excitatory inputs. This was also found to be true in the cat (Sun and May 2014b). The modulatory neuropeptide contained in their dense-core vesicles remains to be determined. Inhibitory (At2 and At2D) terminals to pupil-related motoneurons may arise from the hypothalamic regions that activate pupillary dilation via hypothalamospinal pathways (Sillito and Zbrožyna 1970a, b). The locus coeruleus is also believed to provide an inhibitory input to pupillary neurons in EWpg (Breen et al. 1983; Samuels and Szabadi 2008). This may be part of a system that dilates the pupil with respect to affect, interest and more general activation of the nervous system (Szabadi 2013; Joshi et al. 2016).

We compared the ultrastructure of preganglionic motoneurons that received OPt input, presumably pupillary motoneurons, and other preganglionic motoneurons that did not receive OPt input, a population highly enriched in, but not exclusively, lens-related motoneurons. These two populations share many general features of organization. The pupil-related motoneurons did, however, differ significantly from the mainly lens-related motoneurons in the following respects. Larger numbers of type At2D terminals were observed in contact with the pupillary motoneurons and presumably inhibitory symmetric contacts on pupillary motoneurons were larger. This suggests that pupillary motoneurons receive an additional inhibitory input that is not present on lens-related motoneurons. Perhaps these are tied to decreasing pupillary sphincter tone during dilation. However, while the activity patterns of the lens-related motoneurons have been described in detail (Gamlin et al. 1994), those of the pupil-related motoneurons have not (but see McDougal and Gamlin 2015).

### Pupillary light reflex pathways

**Fig. 16:**
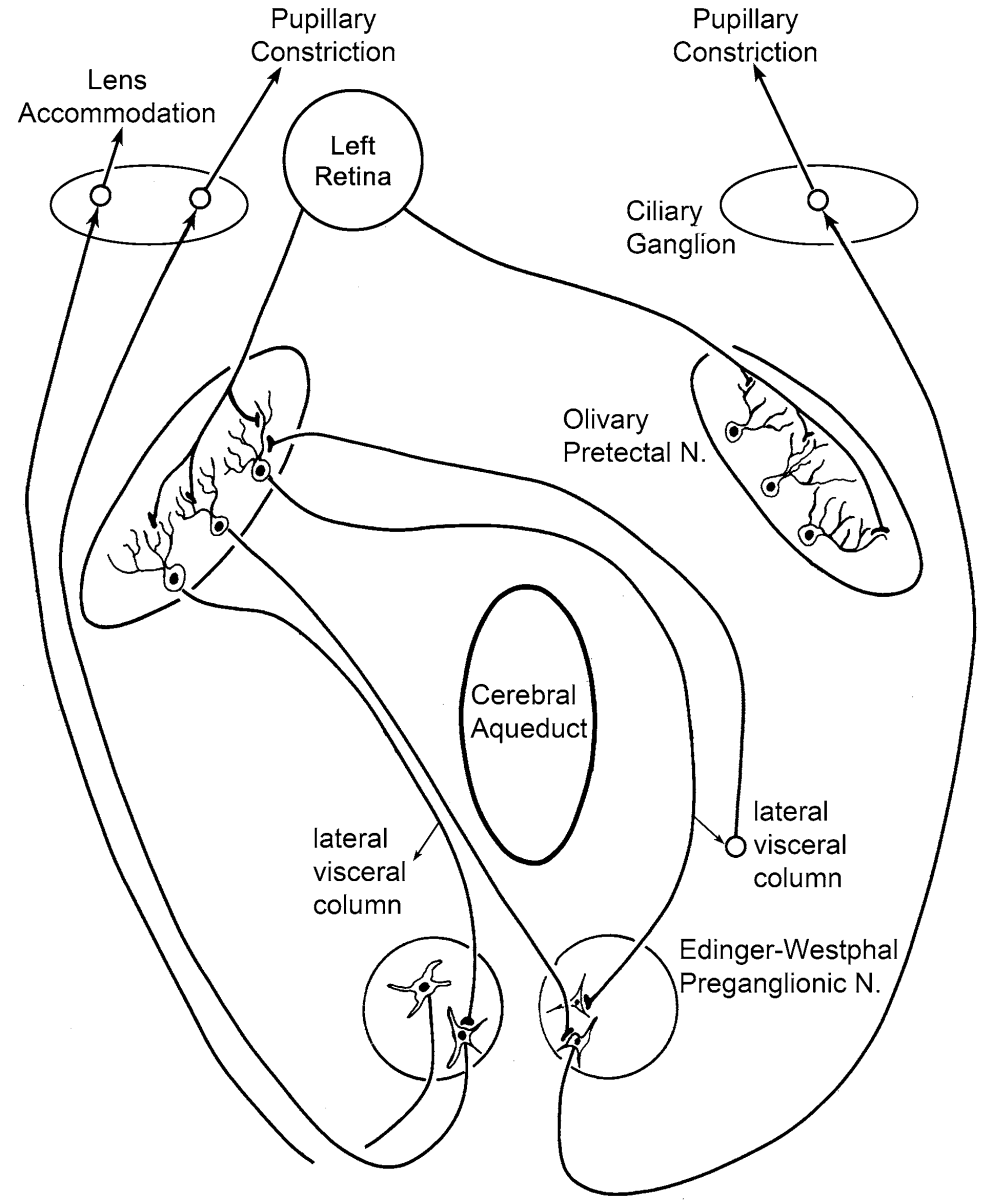
Schematic of pupillary light reflex pathways. The retina projects bilaterally to OPt and synapses directly onto output neurons (only the left eye is shown). OPt output neurons project bilaterally to preganglionic motoneurons in EWpg. These motoneurons project to postganglionic motoneurons in the ciliary ganglion that innervate the sphincter pupillae muscle to produce pupillary constriction. Other preganglionic motoneurons supply the postganglionic motoneurons in the ciliary ganglion that innervate the ciliary muscle to produce lens accommodation. There is also a feedback pathway. The OPt output neurons supply bilateral input to cells in the lvc. These cells project back to contralateral OPt

Taken together, this study and its companion (May and Warren 2019) provide definitive evidence for a pupillary light reflex pathway that involves only two synapses within the central nervous system (Fig. 16). The first synapse is between the axon terminals of intrinsically photoreceptive retinal ganglion cells and projection cells within the OPt. The second is between the terminals of these OPt projection cells and motoneurons in the EWpg. The dual-tracer data presented here for the monkey parallels that presented in the cat (Sun and May 2014b), suggesting that this pattern of connections represents a general mammalian feature. Various other intermediary nuclei, such as the nucleus of the posterior commissure, have been suggested to lie within the pupillary light reflex circuitry (Graybiel and Hartwieg 1974; Berman 1977; Weber and Harting 1980; Breen et al. 1983). While the present data do not eliminate the possibility of additional multisynaptic light reflex circuits relaying through other nuclei, they strongly support a monosynaptic OPt projection to EWpg. Thus, they confirm and extend previous studies indicating a pretectal projection to the EWpg (monkey: Benevento et al. 1977; Steiger and Büttner-Ennever 1979; Gamlin et al. 1995; cat: Itoh 1977; Distler and Hoffmann 1989). OPt projections to EW have also been described in the rat (Itaya and Van Hoesen 1982; Trejo and Cicerone 1984; Klooster et al. 1995), but it is not clear whether these projections terminated in EWpg or EWcp (Smeraski et al. 2004). The nOT has also been suggested as a possible relay for the pupillary light reflex (Büttner-Ennever et al. 1996). Although most terminals were observed in lvc after their pretectal injections, terminations were also charted in SOA, which may have included EWpg. Our data are consistent with a sparse nOT projection, but this may be from the cells related to the near response (Mays et al. 1986).

The present data indicate that the OPt projection to EWpg is a bilateral one (Fig. 16). In fact, it suggests that, in addition to a crossed projection by way of the posterior commissure, the contralateral EWpg also receives input via axons that decussate beneath the cerebral aqueduct. The terminal pattern we observed following OPt injections matches that reported previously in monkeys (Benevento et al. 1977; Büttner-Ennever et al. 1996) and in cats (Distler and Hoffmann 1989; Sun and May 2014b). Some retrograde studies have only reported a contralateral (Steiger and Büttner-Ennever 1979; Clarke et al. 1985a) or predominantly contralateral projection (Gamlin et al. 1995). It is possible that the ipsilateral anterograde labeling we observed here was due entirely to fiber-of-passage uptake. However, we believe this is unlikely, given that the same result was observed with several different tracers (present results), including tritiated amino acids (Büttner-Ennever et al. 1996). Thus, it would appear that morphological substrates for balanced direct and consensual pupillary response are present at the level of the retinal input to OPt and the projection of the OPt to the EWpg.

The area of the EWpg containing motoneurons that had close associations with OPt terminals lay roughly in the middle third of the rostrocaudal extent of the EWpg column. This location differs from that observed in the cat (Erichsen and May 2002; Sun and May 2014b). A simple explanation for this difference might be that the region of EWpg that receives the most contacts in both species lies closest to OPt. Only a portion of the close associations may represent actual synaptic contact. In fact, cells with numerous close associations, like those shown in Fig. 10b, represented quite a small proportion of the motoneuron population. These were found ventrally in EWpg, consistent with a ventral location for pupillary preganglionics reported in marmosets (Clarke et al 1985b, 2003a).

### Lateral visceral column

One of the novel findings described here and in a previous short report (May et al. 2008b) was the presence of a cluster of neurons located dorsolateral to EWpg that projected to the contralateral pretectum. This area received both ipsilateral and contralateral input from the pretectum (Figs. 6 and 7). The location of these cells and terminal fields appears to be the same as that designated as the lvc in three previous studies. In one, trans-synaptic anterograde terminal labeling was seen from retinal injections in macaque monkeys (Kourouyan and Horton 1997). In the others, terminal labeling with a contralateral predominance was observed following pretectal injections (Baleydier et al. 1990; Büttner-Ennever et al. 1996). The designation lvc was used by these authors in the expectation that this nucleus represented one of the EW subdivisions proposed by Burde (1983). As noted above, we have not seen evidence of such subdivisions, and the region in question does not contain cholinergic cells, as a portion of EWpg would (Horn et al. 2008; May et al. 2008a). To maintain some consistency in terminology, we have provisionally maintained the designation lvc, with the proviso that it is not part of EW.

In the companion paper (May and Warren 2019), we described terminals in OPt after injections aimed at EWpg that included lvc. Based on a cross case analysis of our anterograde and retrograde cases, it seems likely that the lvc receives bilateral input from OPt, and that it projects to the contralateral OPt. Moreover, close associations between the pretectal terminals and the projection neurons suggest that this nucleus provides a monosynaptic feedback to OPt. Approximately 40% of the neurons in the primate OPt display bilateral receptive fields that are driven from both eyes (Clarke et al. 2003b). Direct retinal input from the ipsilateral temporal and contralateral nasal retina provides the drive for the contralateral visual field, but central pathways are needed to contribute to the ipsilateral visual field representation. As noted in the companion paper (May and Warren 2019), there is little evidence of commissural OPt connections that could provide this information. Thus, the pattern of connections between the lvc and OPt makes it a good candidate to provide ipsilateral visual field input to the OPt (Fig. 16).

## Abbreviations

3n: Oculomotor nerve
AM: Anteromedian nucleus
At: Axon terminal
At*: Labeled terminal
BC: Brachium conjunctivum
BDA: Biotinylated dextran amine
CC: Caudal central subdivision
Den: Dendrite
Den*: Labeled dendrite
EWpg: Preganglionic Edinger–Westphal nucleus
III: Oculomotor nucleus
InC: Interstitial nucleus of Cajal
lCG: Left ciliary ganglion
lvc: Lateral visceral column
MD: Medial dorsal nucleus
MG: Medial geniculate nucleus
MLF: Medial longitudinal fasciculus
MPt: Medial pretectal nucleus
nD: Nucleus of Darkschewitsch
nL: Nucleus limitans
nOT: Nucleus of the optic tract
nPC: Nucleus of the posterior commissure
OPt: Olivary pretectal nucleus
PAG: Periaqueductal gray
PC: Posterior commissure
PF: Parafascicular nucleus
PPt: Posterior pretectal nucleus
Pul: Pulvinar
R: Red nucleus
rER: Rough endoplasmic reticulum
rCG: Right ciliary ganglion
SOA: Supraoculomotor area
Soma*: Labeled soma
Sp: Spine
WGA–HRP: Wheat germ agglutinin-conjugated horseradish peroxidase
